# Exceptional Concentrations of Gold Nanoparticles in 1,7 Ga Fluid Inclusions From the Kola Superdeep Borehole, Northwest Russia

**DOI:** 10.1038/s41598-020-58020-8

**Published:** 2020-01-24

**Authors:** V. Yu. Prokofiev, D. A. Banks, K. V. Lobanov, S. L. Selektor, V. A. Milichko, N. N. Akinfiev, A. A. Borovikov, V. Lüders, M. V. Chicherov

**Affiliations:** 1 Institute of Geology of Ore Deposits, Petrography, Mineralogy and Geochemistry, Russian Academy of Sciences, Staromonetny per. 35, Moscow, 119117 Russia; 2 School of Earth and Environment, University of Leeds, Leeds, UK; 3 A.N. Frumkin Institute of Physical Chemistry and Electrochemistry RAS, Moscow, 119071 Russia; 4 Faculty of Physics and Engineering, ITMO University, St. Petersburg, 197101 Russia; 5 Sobolev V.S. Institute of Geology and Mineralogy SB RAS, Novosibirsk, Russia; 6 GFZ German Research Centre for Geosciences, Telegrafenberg, 14473 Potsdam, Germany

**Keywords:** Planetary science, Solid Earth sciences

## Abstract

In the drill core of the Kola super-deep borehole (SG-3, 12,262 m depth) gold-bearing rocks of Archaean age have been located at depths of 9,500 to 11,000 m. In veins, between 9,052 and 10,744 m, within this gold zone, quartz contains fluid inclusions with gold nanoparticles. There are 4 types of fluid inclusions (1) gas inclusions of dense CO_2_, (2) liquid-vapor two-phase aqueous inclusions, (3) three-phase inclusions with NaCl daughter crystals, and (4) CO_2_-aqueous inclusions. In all inclusion types, there are extremely high concentrations of gold. The highest gold concentrations were found in the type 3 and 4 fluid inclusions with an average concentration of c. 750 ppm and may be as high as 6,000 ppm. The presence of gold as nanoparticles in the solutions of these fluid inclusions was determined by optical and spectroscopic methods. We suggest that these fluids could be a precursor of “orogenic gold fluids” which, at the gold concentrations determined, would reduce the requirements for large volumes of metamorphic fluids to form orogenic ore deposits. Further, as nanoparticles, gold could be transported in larger amounts than in true solution.

## Introduction

A considerable part of the world gold production comes from deposits classified as “orogenic”. These are predominantly found in fault systems associated with late-stage orogenic crustal growth. They are present in rocks from the Archean to the Tertiary^1–3^. In many instances, the precise age of mineralization is unclear and consequently an exact time relationship with tectonic events is lacking. Therefore, the major issues of how such deposits form particularly with respect to the source of fluid, of metals, of sulphur and depositional processes involved^3–6^ are still unclear. The generation of fluids through metamorphic de-volatilization of hydrous minerals during prograde metamorphism requires orders of magnitude more time (tens of millions of years) than is required for the generation of orogenic vein-hosted mineralization^7^. Mineralization is found largely in retrograde metamorphic terrains, so how then are the fluids preserved in the crust from their generation until they are involved in mineralization? Noteworthy is that worldwide, the composition of fluids associated with orogenic gold mineralization is quite consistent. Fluids are of low salinity, often with a large meteoric component, with significant concentrations of CO_2_ and are therefore quite distinct from the fluids involved in other major styles of gold mineralization such as porphyry or epithermal.

The aim of this work was to study the physicochemical parameters, chemical composition and gold content of the fluids that formed the deep gold mineralization, which was discovered during the drilling of the Kola superdeep well SD-3 at a depth of 9.5–11.0 km. In this study, we have found fluid inclusions trapped at amphibolite facies metamorphic conditions equivalent to an initial depth of ca. 17 km (about 10 km of drill hole and 7 km of erosion section^8^). Different types of fluid inclusion are present, liquid-vapor-halite, liquid-vapor, liquid-vapor-CO_2_ and pure CO_2_ in quartz veins, all containing exceptionally high concentrations of gold and silver.

## Geological Setting

The Kola superdeep borehole (SG-3, 12,262 m) was drilled from 1,970 to 1,992, in the Pechenga ore district of the Russian Federation through approximately a third of the Baltic Shield continental crust. It was the deepest man-made hole on Earth, and was drilled to provide information about the deep structure of the Baltic Shield^8^ and to investigate seismic discontinuities. It goes through the early Proterozoic and Archean complexes composed of volcano-sedimentary rocks, mafic magmatic units and gneisses (Fig. S1). The upper parts of the section, to approximately 4900 m, were metamorphosed to greenschist facies, to 6000 m epidote-amphibolite facies and then amphibolite facies to the bottom of the hole. The Proterozoic regional amphibolite facies metamorphism reached temperatures of 520–650 °C and pressures of 3–4 kbar^8^. Two major fault systems were intersected during drilling: the Luchlompol fault at ca. 4.9 km and an unnamed fault at 10 km^9^. Fluids in aquifers were detected down to 11 km, largely at depths where large fracture zones were intersected.

A gold-bearing zone, with a vertical extension of about 1,500 m, was discovered in the drillcore between 9,500 and 11,000 m. The gold concentration in this zone varies from 0.01 to 6.7 ppm, as was determined by Instrumental Neutron Activation Analysis^8^. The gold mineralization is localized in the metamorphic rocks (two-mica gneisses and amphibolites) of Archean age (2.6–2.8 Ga), that were overprinted by Proterozoic regional epidote-amphibolite and amphibolite facies metamorphism. In polished sections of the core rocks, small flakes (up to 10 μm) and irregularly shaped grains of native gold were observed in biotite, hornblende, plagioclase, and quartz. Gold does not occur within sulphides or in combination with elements other than silver, which can reach up to 26%^10^. Taking into account the erosion, which has cut out about 7 km of the Baltic Shield in the Kola region, the actual depth of the gold mineralized zone was at 16,600–18,000 m during the Proterozoic. Thus, we are dealing with hydrothermal mineralization at depths of the middle crust. Quartz veins containing numerous fluid inclusions are associated with the mineralization, particularly where there are tectonic zones, dated at 1,760 to 1,700 Ma^11^. In the Pechenga area, a greenstone belt of Proterozoic age with several vein and disseminated gold deposits presently outcrops^12,13^.

### Fluid inclusion analyses

**Figure 1:**
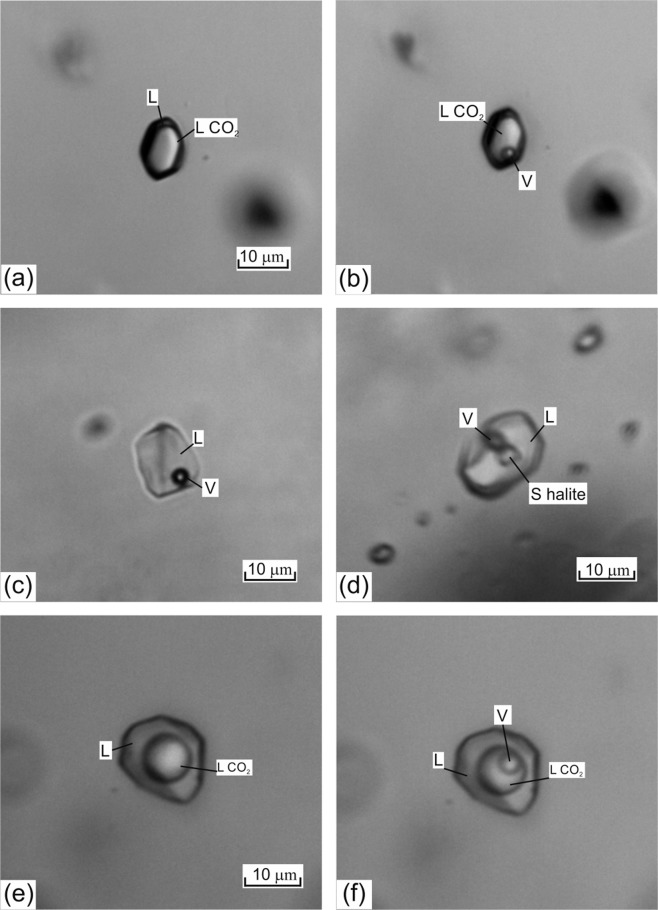
Microphotography of different types of fluid inclusions: (**a**,**b**) – type 1, V – gas inclusions of dense CO_2_ (a +25 °C, b −40 °C), (**c**) – type 2, L-V – two-phase vapor-liquid aqueous inclusions, (**d**) – type 3, L-V-S – three-phase inclusions of chloride brines with NaCl daughter crystals; (**e**,**f**) – type 4, L-LCO_2_ – CO_2_-aqueous inclusions (e +25 °C, f −10 °C). Scale bar 10 µm. Phases: V – gas, L – liquid aqueous solutions, L CO_2_ – liquid CO_2_, S halite – NaCl.

Fluid inclusions in quartz from 9,052 to 10,744 m (the sample numbers correspond to their depth of occurrence) can be divided into four types (Fig. 1): type 1, or V – gas inclusions of dense CO_2_, type 2, or L-V – vapor-liquid two-phase aqueous inclusions, type 3, or L-V-S – three-phase inclusions containing NaCl daughter crystals, and type 4, or L-LCO_2_ – CO_2_-aqueous inclusions. The different inclusion types were studied by microthermometry (Table S1) and Raman spectroscopy (Table S2) to determine the characteristic of the aqueous and gaseous components.

CO_2_-gas inclusions (type 1) homogenized into the liquid phase at temperatures ranging from +30.8 to −45.0 °C. In these fluid inclusions, the melting point of carbon dioxide, TmCO_2_, was between −56.7 and −60.3 °C, and the density varied from 0.37 to 1.14 g/cm^3^. The salinity of the aqueous phase of the gas–rich inclusions was 3.4–4.1 wt. % eq. NaCl. The composition of the gas phase, determined by Raman spectroscopy, was CO_2_ (99.7–98.7 mol. %) and N_2_ (1.3–0.3 mol. %). No CH_4_ or H_2_S was detected in this type of fluid inclusions.

The two-phase fluid inclusions (type 2) are brines containing NaCl, and CaCl_2_ with the eutectic temperatures (Te) from −55 to −74 °C and the ice melting temperature (Tm_ice_) from −20.7 to −63 °C, which corresponds to salinities of 21.6 to 30.2 wt. % eq. CaCl_2_. The homogenization temperature (Th) was between 137 and 228 °C. Raman spectroscopy indicates only low-density N_2_ was present in the gas phase of type 2 fluid inclusions.

Three-phase fluid inclusions (type 3) have halite dissolution temperatures (Th_halite_) from 123 to 381 °C, and the temperatures of homogenization into liquid phase (Th_vapor_) from 137 to 264 °C. The homogenization of vapor phase into the liquid often occurs prior to the dissolution of halite. They also contain NaCl and CaCl_2_ with a Te of −64 °C and have salinities of 25.9 to 45.4 wt. % eq. NaCl, calculated from the dissolution temperature of halite. As with type 2 fluid inclusions, only low-density N_2_ was detected in the gas phase of type 3 fluid inclusions.

CO_2_-aqueous fluid inclusions (type 4) have Th from 203 to 356 °C and salinities of 3.6 to 18.8 wt. % eq. NaCl. Raman spectroscopy shows the composition of the gas phase of type 4 fluid inclusions is very similar to that of type 1 fluid inclusions and includes CO_2_ (99.6–98.1 mol. %) and N_2_ (1.9–0.4 mol. %). Again, no CH_4_ or H_2_S was detected in this type of fluid inclusions.

### Laser ablation inductively coupled plasma mass spectrometry (LA-ICP-MS) analysis of fluid inclusions

**Figure 2:**
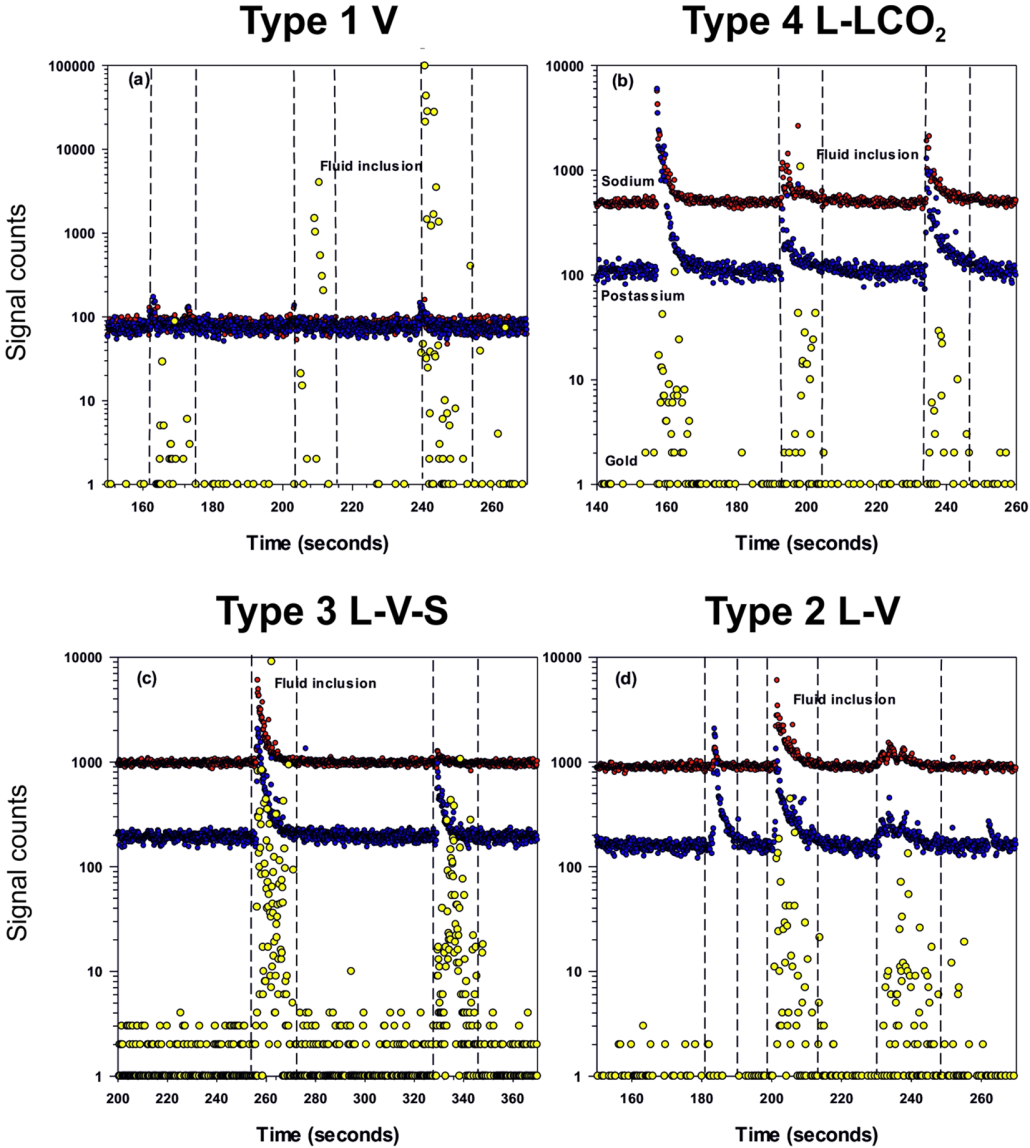
Examples of the LA-ICP-MS signal of different types of fluid inclusions in quartz from the Kola superdeep borehole: (**a**) type 1, (**b**) type 4, (**c**) type 3, (**d**) type 2. Red points – sodium, blue points – potassium, yellow points – gold.

**Figure 3:**
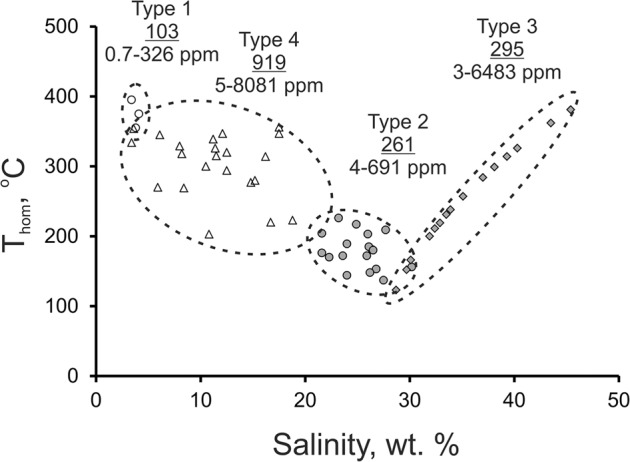
Temperature vs salinity diagram with gold concentrations (mean/min-max) in different types of fluid inclusions.

The analysis of fluid inclusions by LA-ICPMS revealed high gold concentrations in individual fluid inclusions of each of the four types identified, in the veins between 9,500 and 11,000 m (Fig. 2, Supplementary Figs. S2 and S3 and Tables S3 and S4). The gold concentration varies from 7 to 441 ppm (average 103 ppm, n = 14) in type 1 fluid inclusions; from 5 to 690 ppm (average 261 ppm, n = 21) in type 2 fluid inclusions; from 10 to 1,780 ppm (average 295 ppm, n = 24) in type 3 fluid inclusions; and from 27 to 8,081 ppm (average 919 ppm, n = 33) in type 4 fluid inclusions (Table S1). The juxtaposition of these data on the “temperature – salinity” diagram (Fig. 3) shows that maximum concentrations of gold in carbon dioxide-aqueous fluids (type 4) corresponds with a moderate salinity.

The expected LA-ICP-MS ablation signal for dissolved elements in fluid inclusions is a smooth asymmetric shape decreasing in intensity with time. However, the ablation of the four types of fluid inclusions produced a signal for gold consisting of a number of individual spikes, of variable intensity, indicating that the gold was present as particles and not dissolved in fluid. The appearance of the gold signal coincides with that of Na and K which are dissolved in the fluid inclusions, but does not continue after the signal from the inclusion has ceased. Therefore, there is no gold signal when only the quartz is being ablated (Fig. 2), for the four types of fluid inclusions over the entire depth interval investigated.

The large range in measured gold concentrations, which is much greater than the analytical precision, can be caused by the loss of gold particles during the analysis, or by heterogeneous trapping of nanoparticles. This assumes they had already been precipitated from solution, transported and then trapped in the fluid inclusions. Quadrupole ICPMS instruments measure each element sequentially and so a gold particle/s may be transported through the instrument while the ICPMS is measuring another element. In addition, it is entirely possible that different numbers of nanoparticles were trapped in individual inclusions. Therefore, the lowest gold concentrations may be an underestimation, with the real values closer to the highest values measured.

### Presence of nanoparticles

**Figure 4:**
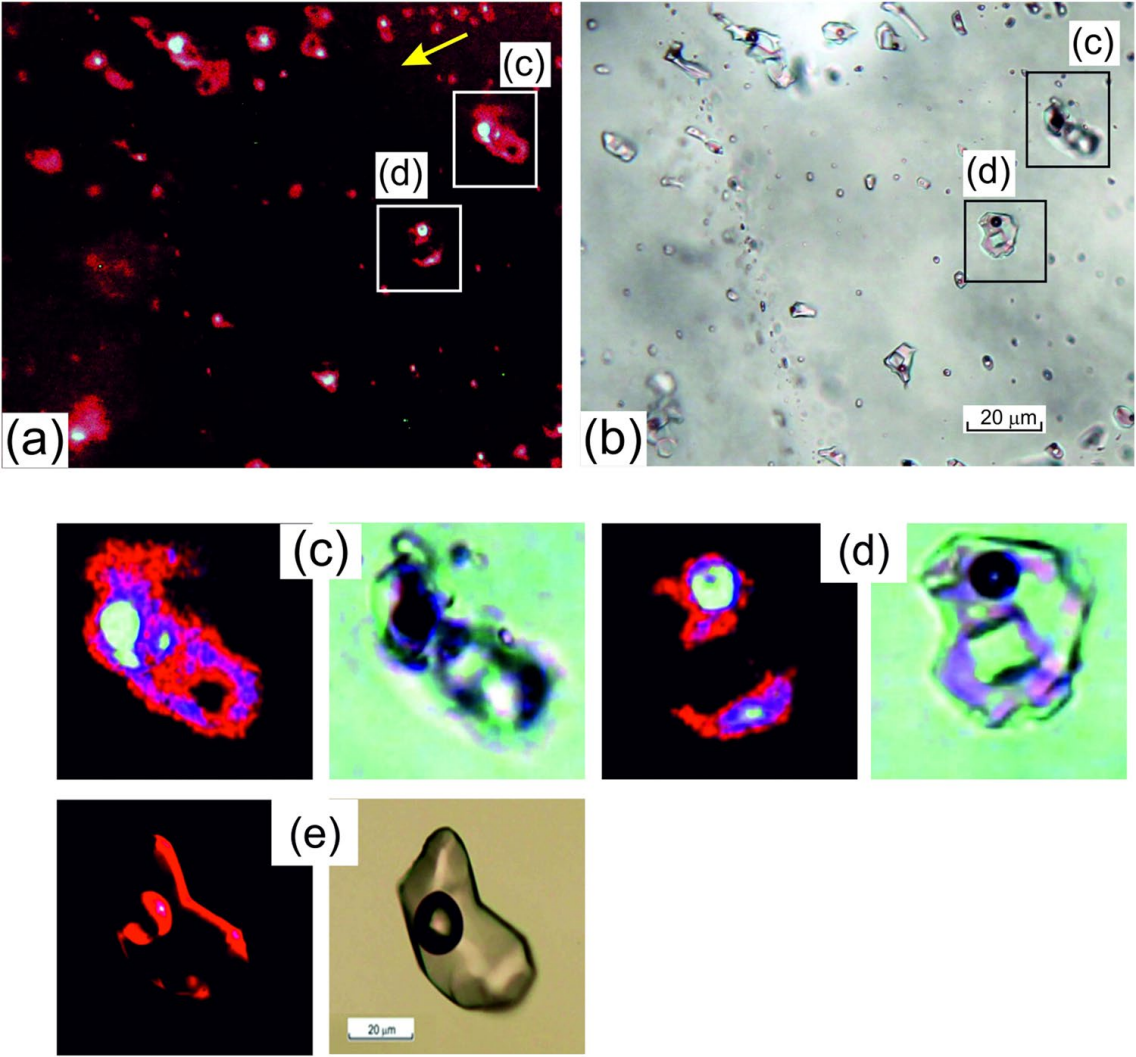
Photomicrographs of the quartz plates with fluid inclusions: the plate with type 3 fluid inclusions from a depth of 9,907 m upon side illumination by red laser beam (**a**), and the same location with transmitted light (**b**); (**c**,**d**) – scaled-up fragments of (**a**,**b**) respectively; and for comparison a plate with a normal (unrelated to this study) fluid inclusion where all components are dissolved (true solution), upon side illumination by red laser beam (**e**, left) and the same location with transmitted light (**e**, right). Side laser illumination of fluid inclusions produces the intense light scattering in the interior solution due to presence of nanoparticles in type 3 inclusions (**a**,**c**,**d**) (red glow of solution inside fluid inclusions and intense white glow around gas bubbles). This is not the case for a true solution (**e**) where light scattering is only observed at interfaces. Scale bar 20 µm.

When the fluid inclusions were ablated the gold signal consisted of a number of spikes rather than the smooth asymmetric signal of elements that are in solution, that indicated gold was present as particles and not in solution (Fig. 2). Similar signal shapes were observed for gold in fluid inclusions at the Arapucandere epithermal deposit, where in open fluid inclusions the 1 µm to 100 nm particulate gold was detected using a scanning electron microscopy (SEM)^14^. In this study particulate gold was not observed in any open fluid inclusions using SEM, suggesting the particles must be much smaller than the limit of detection. However, the presence of much smaller nanoparticles in solutions can be detected as they scatter and reflect monochromatic light. Fluid inclusions were illuminated by laser light in the red part of the spectrum at a wavelength of 657 nm. The laser illumination was at a shallow angle relative to the plane of the quartz plates containing the fluid inclusions (side illumination) and any light reflected from within the fluid inclusions was observed with a normal microscope from above. A plane of L + V + S halite fluid inclusions (Fig. 4a,b) when illuminated by the red laser light (yellow arrow indicates the direction of illumination), have a bright red reflectance from the aqueous portion and a more intense white reflectance from around the vapor bubble. There is no reflectance from the halite crystal. In contrast, when a fluid inclusion unrelated to this study, that does not contain any nanoparticles was illuminated in this manner (Fig. 4e) the only red reflectance was observed from interfaces, i.e., the quartz-liquid and liquid-vapor bubble. There is no red reflectance from the solution contained in this inclusion and no intense white coloration from the vapor bubble. This clearly contrasts with the L-V-halite fluid inclusions of this study (Fig. 4c,d shows a larger scale example of these fluid inclusions). Halite does not reflect any laser light and appears black, while the nanoparticles are homogeneously dispersed in the solution and reflect the red light. However, the intensity of the light reflected is much greater around the vapor bubble and appears white. This indicates that the nanoparticles are present in much greater numbers and are effectively sticking to the surface of the vapor bubble. This also explains why when the fluid inclusions are viewed in plane polarized light they appear as black spheres when normally they would be black around the margins and clear in the center (Fig. 4d). The nanoparticles are producing a metallic sheath on the surface of the vapor bubble which then becomes opaque to plane polarized light, but the lower numbers of particles in the solution allows plane polarized light to be transmitted.

**Figure 5:**
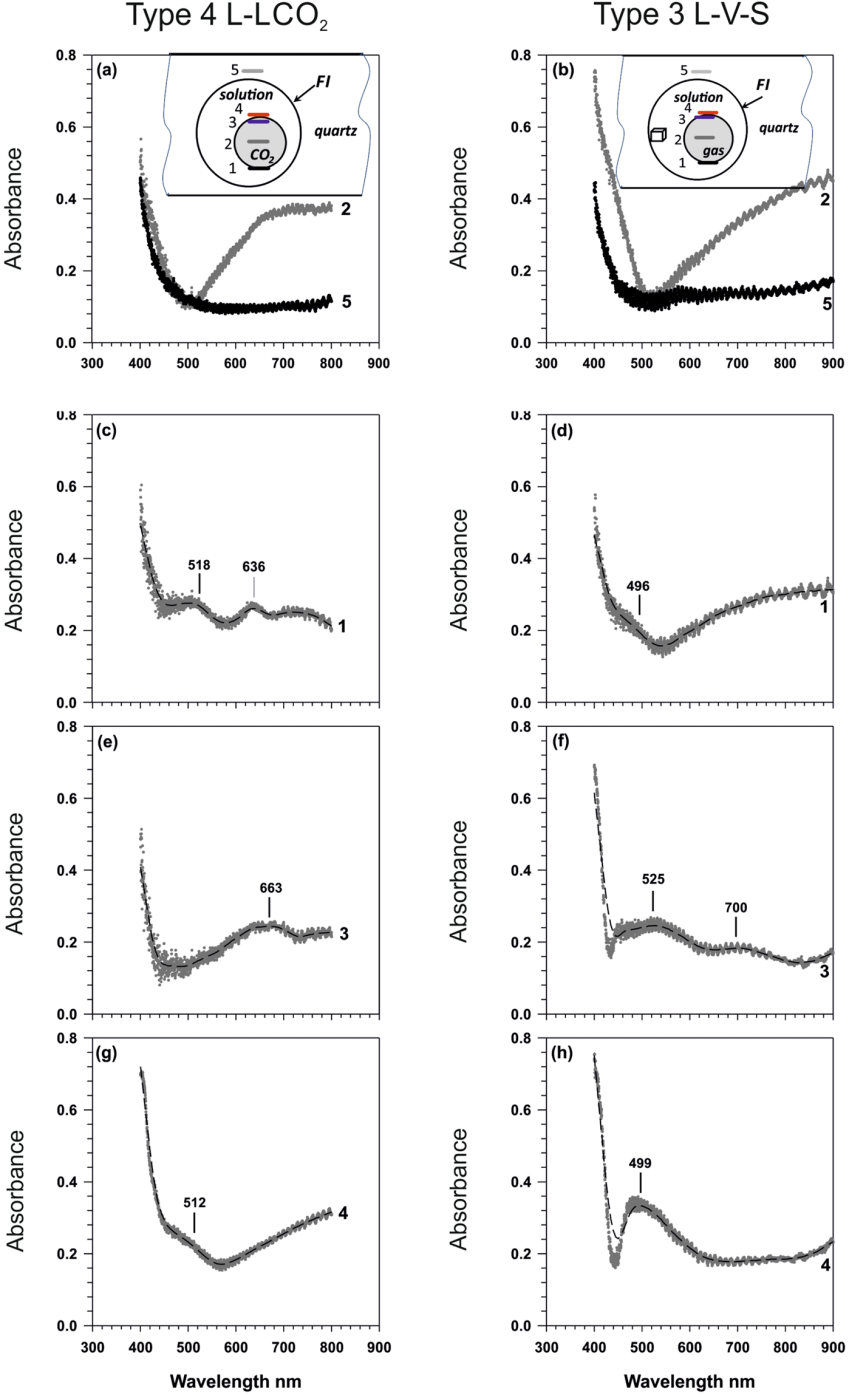
A series of UV-Vis absorption spectra obtained in confocal mode from different locations in type 4 L-LCO_2_ fluid inclusions (**a**,**c**,**e**,**g**) and type 3 L-V-S fluid inclusions (**b**,**d**,**f**,**h**); curve numbers correspond to the positions indicated on the schematic inclusion figures (**a**,**b**).

Additional unequivocal proof of the presence of gold nanoparticles in the fluid inclusions was obtained from the UV-visible absorbance spectra recorded from different areas of the fluid inclusions and the host quartz using confocal microscopy. The system used can obtain spectra from a 1 µm^3^ volume thereby the spectra were recorded from precise areas in the fluid inclusions (Fig. 5).

The λ_max_ for Ag- nanoparticles described in^15^ is 393 nm and for the same size Au- nanoparticles is 520 nm. Intermediate Au-Ag alloy compositions of the same size nanoparticles have intermediate values of λ_max_. As the Au/Ag molar ratio increases, the wavelength at which maximum absorbance is located increases linearly^15^. The size and shape of the nanoparticles also influences the position of the absorbance maxima and the shape of the absorbance spectra. The wavelengths of maxima shift to higher wavelengths of over 650 nm for aggregates of particles of 300 nm but the spectra flatten as the size increases and the peaks are less discernable.

The peaks at 496–525 nm indicate the presence of gold nanoparticles of 18–20 nm in diameter and the bands at 636–700 nm can be attributed to larger aggregates of nanoparticles.

The spectra for two types of inclusions, L + LV CO_2_ and L + V + halite, are shown for different locations in the fluid inclusions (Fig. 5). The spectra for the host quartz and through the center of the vapor bubble (Fig. 5a,b) show no absorbance that is characteristic of nanoparticles. Spectra from the outer edge of the vapor bubble (1), the inner edge of the vapor bubble (3) and in the aqueous portion close to the vapor bubble (4) do reveal absorbance maxima that are associated with the presence of gold nanoparticles. The evidence from LA-ICP-MS analyses is that the nanoparticles are a Au-Ag alloy, which is the form that is usually associated with natural gold. For the type 3 and 4 fluid inclusions the spectra recorded in the solution near the bubble/solution interface have a pronounced band in the region of 520 nm (Fig. 5c,f). This band is characteristic for the plasmonic absorbance of spherical gold nanoparticles of 18–20 nm in diameter^16^. The bands in the region of 495–512 nm (Fig. 5d,g,h) are attributed to Au-Ag alloy nanoparticles. Spectroscopic data was obtained from 12 fluid inclusions with all displaying the same spectral bands shown in Fig. 5. This is univocal evidence of the presence of gold nanoparticles in the aqueous part of the fluid inclusions. There is a greater accumulation of nano-particles near the gas/solution interface, as shown from the more intense reflectance, which is characteristic of such ultra-fine particulate disperse systems. The presence in spectra of an additional absorbance band at 630–700 nm (Fig. 5c,e,f) is attributed to the formation of larger aggregates of nanoparticles. Spectra indicative of nanoparticle aggregates were obtained from the bubble-liquid interface where we have proposed there is a greater accumulation of particles. In the spectra from the center of the vapor bubble and the enclosing quartz there are no absorbance bands that indicate the presence of nanoparticles, thereby confirming that large accumulations of gold nanoparticles are only in the fluid inclusions. Thus, we bring first evidence of deep gold-rich fluids, which contain gold in the form of ultra-fine particles (nanoparticles).

**Figure 6:**
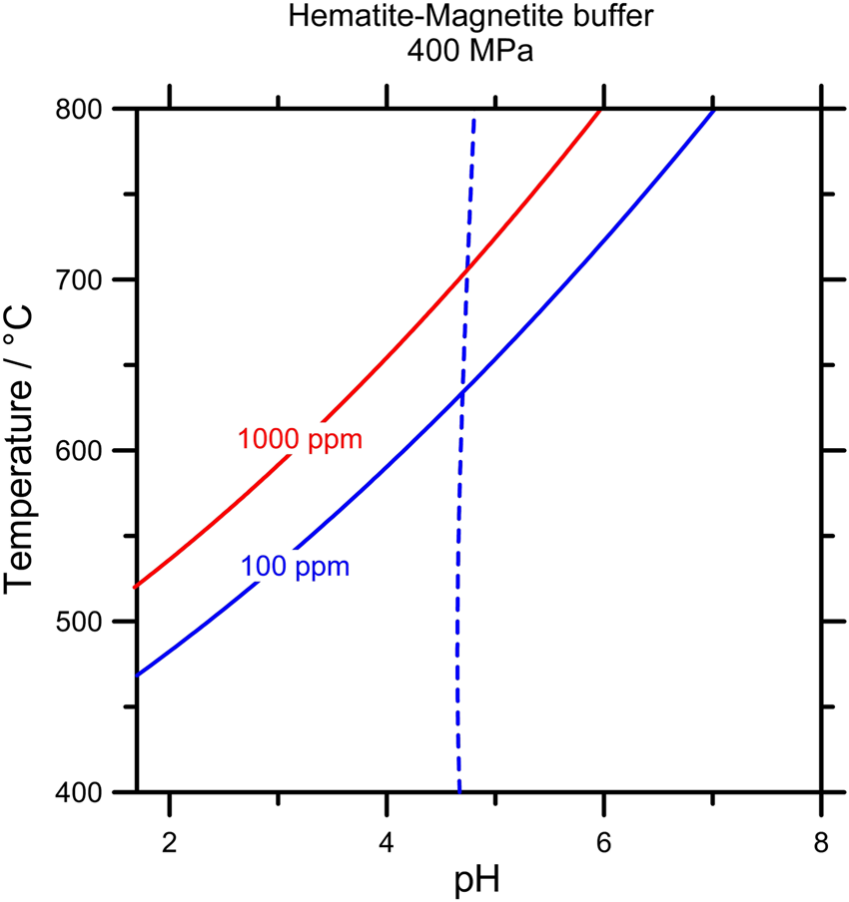
Temperature – pH diagram exhibiting fields where total Au solubility exceeds 100 (blue line) and 1000 ppm (red line). The dotted line indicates the H_2_O neutrality point at given temperature. Computation corresponds to equilibrium with native gold at constant pressure of 400 MPa in the presence of hematite-magnetite mineral buffer and total NaCl content of 32 wt. %.

A thermodynamic simulation of Au solubility was made to estimate the highest Au concentration (as chloride complexes) in these deep fluids, as a function of the temperature and pH. A constant pressure of 400 MPa, a fO_2_ constrained by the hematite-magnetite mineral buffer (hematite and magnetite present in the metamorphic host rocks) and a total NaCl content of 32 weight % (8 mol/kg) were fixed. The data are shown in Fig. 6 (methods section) illustrating the pH – T areas where the total Au concentration in the fluid exceeds 100 (blue line) and 1,000 ppm (red line). The calculations indicate that to reach a maximum Au concentration greater than 1000 ppm at the minimum temperature measured, the fluid must be very acidic. However, if the pH increases it requires an increase of the temperature to achieve the same concentration. Therefore, the temperature of a fluid with the concentration of gold greater than 1,000 ppm exceeds 520 °C for a very acid fluid (pH ≤ 2) and increases to 700 °C at neutral pH. Thus, chloride complexes of gold cannot provide the observed high gold concentrations in aqueous solution with a salinity analogous to natural brines. This suggests that gold had to be present in the fluids as nanoparticles and was not precipitated in the fluid inclusions to form the nanoparticles.

## Discussion

The question is what natural processes caused the formation of fluids containing gold nanoparticles? At some stage gold must have been in solution and solubility varies with pressure-temperature, pH and ligand speciation^17^. Measured concentrations reported in fluid inclusions from the Alpine orogenic belt were primarily between 0.003 and 0.03 ppm which according to thermodynamic modelling is undersaturated by as much as 1,000 times^18^; higher concentrations of 0.5–5 ppm were reported by^19^ but are still very undersaturated. Measurement of gold solubility at saturation in experiments over a range of temperatures confirmed concentrations of over 1,000 ppm were possible^20^.

The data from this study indicate that the fluid inclusions are abnormally rich in gold at mid- crustal depths. Various aspects of gold deposition at depths greater than 15 km have been repeatedly discussed in the literature^21–26^. However, as was noted in^27^ and confirmed by our thermodynamic modeling, the concentration of gold we have measured cannot have been dissolved in hydrothermal fluids at the homogenization temperatures of the fluid inclusions or at higher temperatures, corresponding to the true temperature of trapping, equivalent to the amphibolite grade of metamorphism. In addition, at room temperature the fluid cannot contain such concentrations of gold in solution. It is logical to assume that the gold present in these fluids is in a colloidal (disperse) form, as it is well-known that the concentration of gold as a dispersed phase can be several orders of magnitude higher than its solubility in true aqueous solutions^28^.

Metal nanoparticles are a transitional form between individual metal atoms and more massive crystalline metallic accumulations, comprising hundreds to a few thousand atoms and ranging in size from 1 to 100 nm. Their physical and chemical properties, electrical, magnetic, optical, catalytic, etc., differ from the properties of bulk metals due to their size and internal structure, with almost all the atoms in nanoparticles having the characteristic features of the surface atomic layer. As a result, bulk gold is diamagnetic and does not exhibit any magnetic properties but gold nanoparticles are ferromagnetic^29,30^. If gold can be physically transported as nanoparticles, then much greater concentrations are possible which would require modifications to the models for the formation of gold deposits. The special properties of nanoparticles allow us to consider them as a new form of gold transport in geological processes.

Transport of gold as colloids has been proposed for some time^28,31,32^ as a means of achieving concentrations greater than is possible in solution and has been linked to “bonanza” grades in mineralized veins^31,33^. Direct evidence of gold colloids in hydrothermal fluids, was first reported^34,35^ in fluids from high temperature black smokers exhaled into the cold seawater. However, the first evidence of transportation of colloidal gold particles in and trapped in the fluid inclusions of high temperature ore fluids was from the Arapucandere base-metal ± gold deposit^14^. Cao *et al*.^36^ have also suggested that gold nanoparticles can be transported to the surface in gas from the Earth’s interior.

Type 1–4 fluid inclusions have different vertical distributions within the area of gold mineralization. Dense CO_2_ fluid inclusions (type 1) were found in almost all the samples studied (9,052–10,744 m). Two and three-phase fluid inclusions of brines (types 2 and 3) are predominantly concentrated at depths in the center of the gold bearing zone (9,907–10,583 m). The CO_2_ - H_2_O fluid inclusions (type 4) are located in the shallower part of studied interval at depths from 9,052 m to 10,330 m. The density of carbon dioxide in gas inclusions (type 1) was between 0.37–1.14 g.cm^3^, with the minimum density recorded at a depth of 10,205.8 m. Raman spectroscopy measurements of gas inclusions shows their composition is effectively the same over the whole depth interval at 98.1–99.7 mol. % CO_2_ and 0.3–1.9 mol. % N_2_. The carbon isotope composition of the gaseous inclusions (δ^13^CO_2_ VPDB) gives very homogeneous values of −5.3 to −5.4‰ that corresponds to the mantle value for CO_2_^37,38^. We interpret this to indicate the presence of a stream of deep crustal or mantle carbon dioxide fluid coming from much deeper in the Earth. Type 1 dense CO_2_ inclusions were found throughout the entire borehole depth interval studied, including a depth where other types of inclusions are absent. Therefore, it is unlikely that this dense gas fluid was the product of phase separation of a CO_2_-H_2_O fluid. The evidence is more consistent with a high-temperature gas only fluid enriched in CO_2_, which ascended from deeper in the crust transporting gold nanoparticles. Such a fluid, like other mantle fluids^39^, could carry finely dispersed gold particles in a suspended state. This is consistent with the discovery of gold in the gas fluid inclusions in quartz from the greatest depths, sample 10744, at concentrations from 1 to 281 ppm. According to modern concepts, the subducted slab and sediment wedge or metasomatized mantle lithosphere may have been the source of gold picked up by the CO_2_-rich fluids^26,40^.

If the gold particles are as we propose transported with juvenile CO_2_, how are the saline aqueous fluids containing gold nanoparticles (colloidal gold solution?) formed at elevated temperatures and pressures? The simplest explanation would be to mix an aqueous solution with a gas fluid^41^. Therefore, the disperse colloidal system could have been formed as the result of the interaction of a deep crust or mantle CO_2_ stream carrying gold with the horizon of formational chloride brines that are present between 9,269–10,179 m. The variety of fluid inclusion types in the depth interval from 10,200–9,300 m, which includes the gold-bearing zone, may indicate that it is here the interaction of the different solutions (brines) and CO_2_ occurred. The heterogeneous redistribution of gold nanoparticles between different fluids during their interaction can explain the presence of gold in all types of fluid inclusions. Fluid inclusions of chloride brines (types 2 and 3) from the depth interval of 9,269–10,179 m contain N_2_ in the gas phase without any CO_2_. Since the gas phase in these fluid inclusions separated from the brines upon cooling, the absence of CO_2_ can be the result of its very low solubility in saline brines. Therefore, we assume that the gas-rich inclusions (type 1) and the brines in other fluid inclusions (types 2 and 3) have different sources. Most likely, the high salinity fluids occurred in a horizon where formational brines were present. The formation of such brines and their relation to metamorphic fluids has previously been discussed^42^.

**Figure 7:**
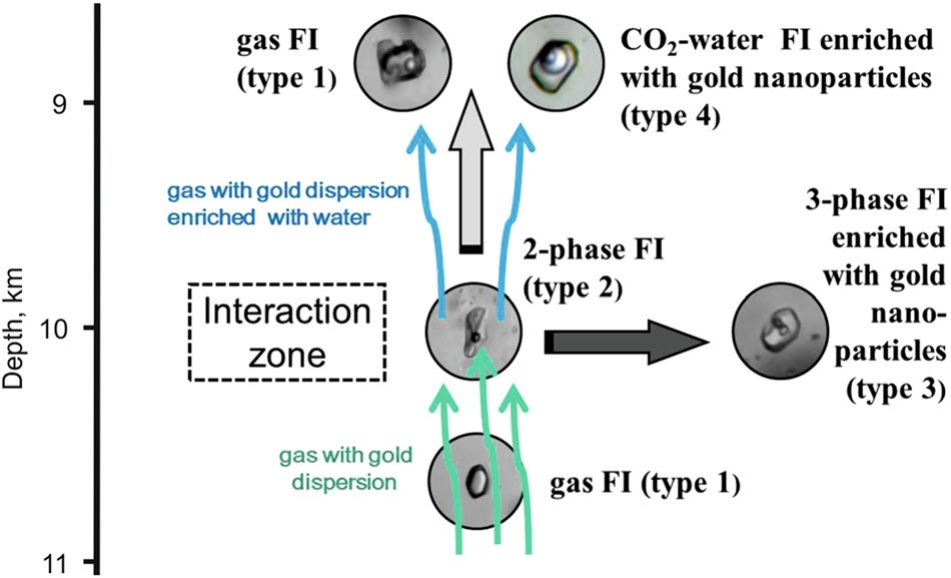
The hypothetical scheme for the accumulation of gold nanoparticles in aqueous fluids by interaction of the mantle CO_2_ fluid with formation brines present in pores in lower part of the earth’s crust.

The process of an effective interaction between a CO_2_ gas stream and a horizon of saline waters was shown to have occurred at the Darasun goldfield^43^. We propose that a similar process is confirmed by the presence of a minimum CO_2_ density at a depth of 10,205.8 m, i.e., in the deeper part of the inferred brine horizon. Thermodynamic modelling of mixing CO_2_ and an aqueous chloride solution has shown^41^ that during interaction part of the CO_2_ dissolves in the aqueous solution, while part of the water passes into the CO_2_ gas-phase, leading to an increase of the chloride concentration in the dominantly aqueous fluid. The interaction also produces a large number of gas bubbles, that is, the appearance of a large gas-liquid interface area per unit volume of the system. This leads to the initiation of various kinds of surface phenomena with high coefficients of phase-to-phase energy and matter transfer. Such processes of interphase interaction may result in the transition of gold (and probably other metals) from the gaseous carbon dioxide phase into the aqueous fluid and its accumulation there in the form of nanoparticles (Fig. 7).

The involvement of CO_2_-rich low salinity aqueous fluids is a consistent feature of orogenic gold deposits, but the involvement of CO_2_ without an aqueous component is much rarer and fluids are predominantly of much lower salinity. Studies of the fluid inclusions from the Ashanti deposit in Ghana, by^44,45^ resulted in the interpretation that the gold was transported by aqueous-free carbonic fluids. Klein and Fuzikawa^46^ noted the involvement of CO_2_-only fluids from charnockites in the transport of gold at the Carara deposit in Brazil. Likewise^47^, suggested gold transport by carbonic fluids for deposits hosted in amphibolite-granulite rocks in the Limpopo belt of South Africa. While most orogenic deposits conform with a source from continental metamorphic fluids^26^, points out that hypozonal, amphibolite deposits do not. The model they proposed for the Jiaodeng deposit, applicable to other provinces, is one of slab de-volatilization. Over-pressured auriferous fluids ascend into deep crustal faults and then directly to the site of mineralization. The higher CO_2_ content fluids interact with anomalous gold bearing minerals resulting in higher gold concentrations. We suggest the fluid inclusions in this study, containing very high gold concentrations, are examples of such fluids.

The area of the earth’s crust under consideration corresponds to the depths of the middle crust and, according to modern concepts, conforms to the level of mobilization of fluids of orogenic gold deposits^3^. Current estimates of gold concentration in fluids of orogenic deposits are 0.5–5 ppm^19^. This is approximately 2–3 orders of magnitude lower than the maximum concentration of gold in the fluids from the Kola superdeep borehole. The discovery of gold nanoparticles in these fluids prompts a new look at the scale of the migration of gold and the ratio of the masses of the fluid and the metal carried by it. Gold was transported from deeper in the crust, than we were able to sample, as particulates by CO_2_ fluids and are only present in aqueous fluids when these are intersected by CO_2_. If this is indeed the case, then it provides an alternative to the generation of metamorphic aqueous fluids deep in the crust as a requirement to transport gold to sites of mineralization.

## Conclusions

The extremely high concentrations of gold in fluid inclusions of chloride brines and CO_2_-rich fluids were determined. The concentrations are much greater than previously thought possible as a consequence of gold being in the form of nanoparticles. The presence of gold nanoparticles in fluid inclusions was confirmed by optical and spectroscopic analysis. Based on these results we might assume that there are reservoirs of fluids containing gold nanoparticles in the Earth’s lithosphere that can be a source of Au-bearing mineralizing fluids for the formation of gold deposits. Therefore, the data obtained significantly broadens our understanding of the boundary parameters of the deep processes of transport and accumulation of gold by hydrothermal fluids. Further study of these newly discovered fluids will expand of our understanding of the gold behavior in deep fluid systems and mechanism of its accumulation in the Earth’s crust.

## Methods

### Fluid inclusion microthermometry

Double-polished 0.3–0.5 mm thick sections were made from quartz samples for visual, thermometric, and cryometric studies of fluid inclusions. Fluid inclusions were studied using an Olympus BX51 optical microscope. Microthermometry of fluid inclusions was performed with a measuring complex consisting of a THMSG-600 heating stage (Linkam, United Kingdom) attached to the Olympus microscope equipped with long-focal-length lenses, a video camera, and a control computer. The salt compositions of solutions were determined from eutectic temperatures^48^. Fluid salinity was estimated from ice melting temperatures of two-phase inclusions and/or from homogenization temperatures of halite daughter crystals in multi-phase inclusions in the NaCl–H_2_O system^49^. Fluid salinity in CO_2_–water inclusions was estimated from gas hydrate melting temperatures^50^. Carbon dioxide concentrations in solutions were calculated from the volume and weight ratios of fluid components^51^.

In cases where minerals contained co-genetically trapped CO_2_–water and CO_2_ gas inclusions, pressure was determined from the intersection of the isochore with the isotherm^52^. Fluid inclusions that trapped heterogeneous fluids within two-phase equilibrium line, do not require pressure corrections and, homogenized at the highest temperature^53^.

Salt concentration, fluid density, and fluid pressure were calculated using the FLINCOR software Version 1.2.1. for Windows (the next versions were made for Apple)^54^.

### Raman spectroscopy

Compositions of the gas and solid phases of fluid inclusions were studied by Raman spectroscopy in IGM SB RAS (V.S. Sobolev Institute of Geology and Mineralogy, Siberian Branch of the Russian Academy of Sciences, Novosibirsk) using a Jobin Yvon LabRAM HR800 spectrometer.

### Laser ablation ICP-MS of fluid inclusions and quartz

The chemical analysis of individual inclusions, or groups of small related inclusions, was made by Laser-ablation inductively-coupled mass-spectrometry (LA-ICPMS) using an Agilent 7500c mass spectrometer, combined with a Geolas Q Plus laser ablation system^55^. This system uses a Compex 103 ArF excimer laser producing a wavelength of 193 nm with an energy density typically 10 Jcm-2 at the sample surface. The operating frequency of the laser was 5 Hz with spot sizes of 25 µm and occasionally 50 µm, the main criteria being that the size was greater than that of the inclusions. The ablated material was transported from the ablation cell to an Agilent 7500c ICP-MS using 99.9999% He flowing at 2 ml min-1 into a cyclone mixer where it was combined with the Ar carrier gas flowing at 1.02 ml min-1. The mixer prolongs the signal from the ablated inclusions and improves precision by increasing the number of cycles through the selected elements and therefore the number of determinations of their ratio relative to Na. The instrument was operated in reaction cell mode using 2.5 ml min-1 99.9999% H2 to remove interferences from 40Ar on 40Ca and from 56ArO on 56Fe. For the analysis of fluid inclusions element/Na intensity ratios were converted to weight/weight ratios using the NIST glass standard SRM-610 and the soda lime standard SRM-1412 (for K/Na ratios that were close to 1) for calibration. For the analysis of quartz 50 µm ablation spots were used and the elements determined against Si as the internal standard element. NIST SRM-610 was used to check for instrumental drift, which was insignificant over each day’s analysis. Integration of the standard and sample signals used the SILLS, version 1.0,1, software package^56^. Full details of the analytical protocols and calibration of the instrument are presented in^57^.

The following elements were analyzed in two different ablation programs, Li, Na, Mg, K, Ca, Mn, Fe, Cu, Zn, Sr, Ag, Ba and Pb in one and N, K, Ag, Sb Au in the other. The results were combined to give the overall fluid composition. In addition, the program with the reduced element list had increased dwell times of 20 ms for Ag and 50 ms for Au, other elements were all measured for 10 ms. The increased time obtaining signal from the Au and Ag was due to these elements (especially Au) being present as particles and not in solution. As the ICP-MS has a quadrupole system each isotope is measured sequentially and while measuring an isotope of another element particulate gold may be passing through the ICP without being detected. Therefore, having a longer measurement time gives better accuracy.

In sample 9907 3 types of fluid inclusions were found that represent the same types from other sample depths (L-V-H, LV and V-L). Analyses of sample 9907 sho that Na, Mg, K, Ca were the major fluid components so together with Ag and Au these were the elements measured in samples 9052, 9269, 10179 and 10331. All element weight ratios relative to the internal standard element are presented in the supplementary data.

### Spectral measurement of fluid inclusion nano-particles

Using the confocal scanning microscopy, we measured the local transmission spectra of individual portions of the fluid phase, with a volume of 1μm^3^, located inside the fluid inclusion. The fluid inclusions studied were located in plane-parallel quartz plates of 0.4 mm thickness. These plates were placed on a 3-axis stage (Thorlabs, MBT616D/M). This allowed for precise moving the inclusions along the z-axis for analysis the transmission spectra from individual portions at different locations (Fig. 5a,b). The fluid inclusions were irradiated by white light (halogen lamp HL2000 FHSA Ocean Optics), and the transmitted optical signal was collected by an objective (Mitutoyo M Plan APO HR 100×, 0.9 NA) and then analyzed by commercial confocal spectrometer Horiba LabRam HR with a cooled CCD camera (Andor DU 420A-OE 325) and 600 g/mm diffraction grating. The spectra of the white lamp and the optical path without the inclusions were measured in advance for normalization the signal transmitted through the sample. Conversion of the measured transmission spectra T to absorption A (Fig. 5) was performed mathematically (A = 1–T) assuming that the scattering and reflection from the gold nanoparticles and surface roughnessdo not significantly affect the absorption spectra.

### Carbon isotopic determination of fluid inclusion gases

Carbon isotopic composition of fluid inclusion gases were measured at the GFZ Potsdam, Germany using a sample crusher, GC-column, EA, a ConFloIII interface, and a Thermo DeltaplusXL mass spectrometer according to the online method described by Plessen and Lüders^58,59^. These measurements were carried out using VPDB standard. Variations in δ^13^C values of CO_2_ of quartz-hosted gas-rich fluid inclusion assemblages in previously studied quartz chips from the Ashanti gold mine (GH-172 and GH-151^58^, show excellent reproducibility in the range between 0.5 and 0.4‰, respectively. This implies that the δ^13^C CO_2_ values of the analyzed fluid inclusion gases were dominated by the supply of CO_2_ during a major fluid event and that the CO_2_-rich fluid inclusions are probably representative for characterizing the origin of the Au-bearing fluids^38^.

### Thermodynamic simulations

The thermodynamic modeling was carried out using the Gibbs free energy minimization algorithm implemented in the HCh program (version 4.5 for Windows)^60,61^. Thermodynamic data for Au aqueous complexes were adopted from the study of^62^, with the exception of data for gold dichloride complex, AuCl_2_^–^, that was adopted from the recent study of^63^. Data for minerals and other aqueous species are from the SUPCRT database^64^.

An extended Debye–Hückel expression^65^ was used to calculate individual molal activity coefficients for the species i with charge zi:

$$\log \,{\gamma }_{i}=-\,\frac{A{z}_{i}^{2}{I}^{0.5}}{1+B{\dot{a}}_{i}{I}^{0.5}}+{B}_{\gamma }I+\varGamma ,$$

where *A* and *B* are the Debye–Hückel solvent parameters taken from^65^; the distance of closest approach of ion i, is given a value of 4.5 Å for å for all ions; *I* is the effective ionic strength using the molal scale; *Γ* is a mole fraction to molality conversion factor; and *B*_*γ*_ is the extended-term parameter (b-dot coefficient) taken from^66^ for NaCl. Figure 6 illustrates pH – T areas where total Au concentration in the fluid exceeds 100 (blue line) and 1000 ppm (red line). It can be seen that to reach the utmost Au content at the least temperature fluid should be acid, while the increasing pH brings to increasing the least temperature. In particular, temperature of the fluid with c(Au) > 1000 ppm should exceed 520 °C for the very acid fluid and 700 °C for the neutral one.

## Supplementary information

Supplementary information.
