# Supplementary material for: Exceptional Concentrations of Gold Nanoparticles in 1,7 Ga Fluid Inclusions From the Kola Superdeep Borehole, Northwest Russia
Source: Sci Rep. 2020 Jan 24;10:1108. doi: 10.1038/s41598-020-58020-8 (PMC6981266; doi:10.1038/s41598-020-58020-8)
Supplement: Supplementary file 1 — Supplementary information. [file 41598_2020_58020_MOESM1_ESM.doc]

# Supplementary Figures and Data


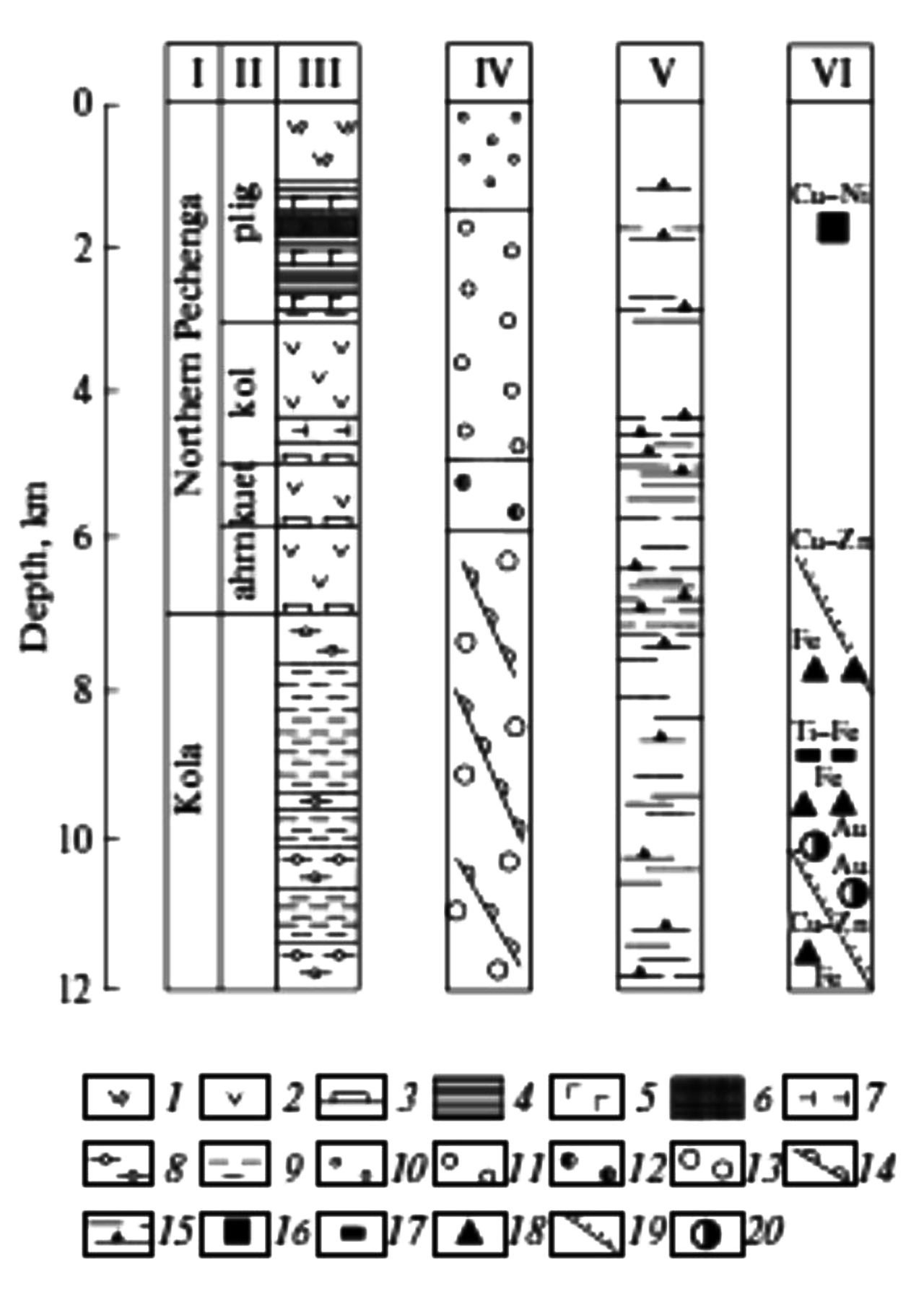


Fig. S1. The position of ore mineralization in the section of the Kola Superdeep Borehole [2].

I, Series of rocks;

II, suites of the Early Proterozoic Northern Pechenga series (pilg–Pilgujarvi, kol–Kolosjoki, kuet–Kuotsjarvi, ahm– Ahmalahti);

III, geological section;

IV, metamorphic facies;

V, zone of plastic deformations;

VI, ore mineralization;

(1) metabasalts with interlayers of picrite; (2) metabasalts; (3) metasandstones; (4) metasedimentary rocks of the productive formation; (5) gabbro; (6) peridotites; (7) metaandesites; (8) high-clayey gneiss; (9) biotite– amphibole gneiss; (10–14) metamorphic facies: (10) prenitpumpelyite, (11) greenschist, (12) epidote-amphibolite, (13) amphibolite, (14) greenschist zones; (15) cleavage zones; (16–20) ore mineralization: (16) copper-nickel, (17) iron–titanium, (18) iron quartzites, (19) polymetallic hydrothermal, (20) aurum–argentum.

**Table S1.** The parameters of the mineralizing fluids in fluid inclusions in quartz of veins of SG-3 superdeep borehole.

| Sample depth (m) | Incl. Type * | n | *T*h ºC | *T*eut ºC | *T*m ice ºC  (or Td NaCl) | *T*m CO_2_ ºC | *T*h CO_2_ ºC | *T*m Clathrate ºC | Eq. mass% NaCl or  (CaCl_2_) | CO_2_ mol/kg solution | *d,* g/cm^3^ |
| --- | --- | --- | --- | --- | --- | --- | --- | --- | --- | --- | --- |
| 9052.6 | 4 | 11 | 356 - 347 | **-**42 - **-**41 | **-**11.9 - **-**11.1 | **-**58.5 - **-**58.2 | +7.0 - +9.6 (L) | **-**2.2 - **-**1.8 | 17.9 - 17.5 | 6.9 - 6.5 | 1.05 |
|  | 1 | 55 | **-** | **-** | **-** | **-**60.3 - **-**56.8 | **-**12.0 - +22.4 (L) | **-** | **-** | **-** | 0.75 - 0.99 |
|  | 4 | 2 | 203 | **-**34 | **-**3.0 | n.d. | +25.0 (L) | 3.8 | 10.8 | 2.6 | 1.04 |
| 9269.4 | 4 | 6 | 354 | **-**36 | **-**4.1 | **-**57.7 | +30.2 ( L) | 8.2 | 3.6 | 6.1 | 0.92 |
|  | 1 | 12 | **-** | **-** | **-** | **-**57.6 - **-**57.0 | **-**2.8 - +18.5 (L) | **-** | **-** | **-** | 0.94 - 0.79 |
|  | 3 | 14 | 175 - 146 | n.a. | (284 - 200) | **-** | **-** | **-** | 37.0 - 31.9 | **-** | 1.09 - 1.12 |
|  | 2 | 43 | 144 - 226 | **-**64 - **-**67 | **-**22.4 - **-**34.8 | **-** | **-** | **-** | (22.3 - 26.2) | **-** | 1.02 - 1.12 |
| 9907.5 | 3 | 30 | 185 -259 | **-**64 | (231 - 123) | **-** | **-** | **-** | 33.5 - 28.7 | **-** | 1.11 - 1.16 |
|  | 2 | 56 | 185 - 137 | **-**74 - **-**64 | **-**63.0 - **-**33.0 | **-** | **-** | **-** | (30.2 - 25.9) | **-** | 1.09 - 1.15 |
|  | 4 | 33 | 347 - 300 | **-**30 - **-**35 | **-**2.6 - **-**12.5 | **-**57.0 - **-**57.2 | +8.9 - +27.5 (L) | 2.9 - 8.3 | 3.4 - 12.1 | 6.3 - 7.0 | 0.92 - 1.03 |
|  | 1 | 185 | **-** |  | **-**4.3 - **-**3.1 | **-**58.9 - **-**57.1 | **-**6.1 - +22.7 (L) | 8.3 - 7.9 | 4.1 - 3.4 | **-** | 0.96 - 0.74 |
| 10179.1 | 3 | 12 | 228 - 200 | n.a. | (381 - 314) | **-** | **-** | **-** | 45.4 - 39.3 | **-** | 1.24 - 1.09 |
|  | 2 | 28 | 217 - 172 | **-**68 - **-**64 | **-**33.2 - **-**20.7 | **-** | **-** | **-** | (26.0 - 21.6) | **-** | 1.02 - 1.08 |
| 10205.8 | 1 | 234 | **-** | **-** | **-** | **-**59.3 - **-**57.3 | 30.5 - **-**30.3 (V-L) | **-** | **-** | **-** | 0.37 - 1.08 |
|  | 4 | 17 | 314 - 277 | **-**60 - **-**30 | **-**12.8 - **-**4.6 | **-**59.3 - **-**57.5 | +3.4 - **-**17.5 (L) | 4.5 - 5.2 | 16.3 - 14.8 | 5.3 - 3.1 | 1.09 |
| 10331.2 | 4 | 49 | 345 - 269 | **-**46 - **-**36 | **-**6.7 - **-**3.7 | **-**57.0 - **-**56.7 | +2.5 - +25.0 (L) | 2.6 - 5.4 | 12.5 - 8.4 | 7.1 - 4.9 | 0.98 - 1.04 |
|  | 1 | 20 | **-** | **-** | **-** | **-**57.1 - **-**56.7 | **-**1 - +27.7 (L) | **-** | **-** | **-** | 0.66 - 0.94 |
|  | 4 | 4 | 223 | **-**42 | **-**16.6 | n.a. | +30.8 ( L) | n.d. | 18.8 | 1.3 | 1.12 |
| 10583.5 | 1 | 72 | **-** | **-** | **-** | **-**57.8 - **-**57.2 | **-**45 - +4.2 (L) | **-** | **-** | **-** | 1.14 - 0.90 |
| 10690.0 | 1 | 40 | **-** | **-** | **-** | **-**57.8 - **-**57.1 | **-**15 - +12.9 (L) | **-** | **-** | **-** | 1.01 - 0.84 |
|  | 3 | 6 | 264 - 180 | n.a. | (299 - 238) | **-** | **-** |  | 38.1 - 33.9 | **-** | 1.12 - 1.16 |
| 10744.9 | 1 | 98 | **-** | **-** | **-** | **-**58.2 - **-**57.4 | **-**21.4 - **-**8.9 (L) | **-** | **-** | **-** | 1.04 -0.98 |

Note: Inclusions Type: 1 – vapor CO_2_, 2 – gas-liquid water-salt solutions, 3 – chloride brines. 4 – CO_2_-water-solutions. n – number of inclusions. L – homogenization of CO_2_ in liquid, V – in gas,. d – density of fluid.

**Table S2.** The composition of gas phase of different types fluid inclusions (Raman spectroscopy, mol. %).

| Sample depth inclusion | Inclusion type | CO_2_ | N_2_ | H_2_S | CH_4_ |
| --- | --- | --- | --- | --- | --- |
| 9052,6 (2) | V | 98.7 | 1.3 | 0.0 | 0.0 |
| 9052,6 (4) | V | 99.6 | 0.4 | 0.0 | 0.0 |
| 9907,5 (3) | V | 99.7 | 0.3 | 0.0 | 0.0 |
| 9907,5 (7) | V | 99.7 | 0.3 | 0.0 | 0.0 |
| 10205,8 (1) | V | 99.5 | 0.5 | 0.0 | 0.0 |
| 10205,8 (2) | V | 99.5 | 0.5 | 0.0 | 0.0 |
| 10331,2 (3) | V | 99.5 | 0.5 | 0.0 | 0.0 |
| 10583,5 (1) | V | 98.9 | 1.1 | 0.0 | 0.0 |
| 10583,5 (2) | V | 98.9 | 1.1 | 0.0 | 0.0 |
| 10583,5 (3) | V | 99.2 | 0.8 | 0.0 | 0.0 |
| 10744,9 (1) | V | 99.5 | 0.5 | 0.0 | 0.0 |
| 10744,9 (2) | V | 99.4 | 0.6 | 0.0 | 0.0 |
| 9907,5 (4) | L-V | 0.0 | 100.0 | 0.0 | 0.0 |
| 9907,5 (5) | L-V | 0.0 | 100.0 | 0.0 | 0.0 |
| 9907,5 (6) | L-V | 0.0 | 100.0 | 0.0 | 0.0 |
| 9907,5 (1) | L-V-H | 0.0 | 100.0 | 0.0 | 0.0 |
| 9907,5 (2) | L-V-H | 0.0 | 100.0 | 0.0 | 0.0 |
| 9052,6 (1) | CO_2_-aqua | 99.4 | 0.6 | 0.0 | 0.0 |
| 10205,8 (3) | CO_2_-aqua | 98.1 | 1.9 | 0.0 | 0.0 |
| 10331,2 (1) | CO_2_-aqua | 99.5 | 0.5 | 0.0 | 0.0 |
| 10331,2 (2) | CO_2_-aqua | 99.6 | 0.4 | 0.0 | 0.0 |

**Table S3.** Calculated Ag and Au concentrations in fluid inclusions from different depths (m)

| **9052** | | **9269** | | **10179** | | **10331** | | **10744** | |
| --- | --- | --- | --- | --- | --- | --- | --- | --- | --- |
| **V-L** | | **L-V-Halite** | | **L-V-Halite** | | **LV CO_2_+L** | | **V-L** | |
| Ag ppm | Au ppm | Ag ppm | Au ppm | Ag ppm | Au ppm | Ag ppm | Au ppm | Ag ppm | Au ppm |
|  |  |  |  |  |  |  |  |  |  |
| 240 | 628 | 171 | 1710 | 7 | 61 |  | 89 | 56 | 3 |
| 29 | 94 | 257 | 2431 | 67 | 307 |  | 42 | 54 | 3 |
|  | 493 |  | 2571 |  | 425 |  | 12 | 48 | 24 |
|  | 270 | 534 | 45781 |  | 70 |  | 5 | 65 | 3 |
|  | 252 |  | 76 | 224 | 6483 | 20 | 165 | 61 | 3 |
| 34 | 293 |  | 76 | 1007 | 3498 |  | 34 | 33 | 6 |
|  | 1088 |  | 421 |  | 146 | 3 | 75 | 47 | 2 |
| 25 | 190 | 318 | 640 | 17 | 139 | 286 | 2078 | 4389 | 109 |
|  | 107 | 171 | 1710 | 103 | 1606 | 201 | 427 | 33 | 2 |
| 37 | 301 | 257 | 2431 | 396 | 306 | 271 | 1093 | 419 | 281 |
|  | 705 |  | 2571 | 31 | 109 | 137 | 2140 | 22 | 16 |
| 35 |  | 534 | 45781 | 13 | 3 | 677 | 971 | 6 | 3 |
| 91 | 121 |  | 76 | 44 | 340 | 728 | 997 | 1896 | 33 |
|  | 78 |  | 76 | 31 | 3 | 122 | 438 | 26 | 10 |
| 6 | 31 |  | 421 | 13 | 55 | 16 | 154 | 63 | 96551 |
| 93 | 520 | 318 | 640 | 80 | 224 | 73 | 190 | 24 | 10 |
| 71 |  | 171 | 1710 | 27 | 1881 | 353 | 1810 | 23 | 1225 |
| 34 | 31 | 257 | 2431 | 14 | 63 | 400 | 3874 | 12 | 1 |
| 77 | 354 |  |  | 7 | 61 | 32 | 380 | 27 | 34 |
| 56 | 310 |  |  | 67 | 307 | 100 | 191 | 14 | 1 |
| 22 | 206 |  |  |  | 425 | 113 | 3302 | 353 | 264 |
| 21 | 207 |  |  |  | 70 |  |  | 13 | 1 |
| 55 | 355 |  |  |  |  |  |  | 10 | 248 |
| 19 | 1383 |  |  |  |  |  |  | 36 | 111 |
|  |  |  |  |  |  |  |  | 50 | 3 |
|  |  |  |  |  |  |  |  | 2992 | 2371 |
|  |  |  |  |  |  |  |  | 7 | 10 |
|  |  |  |  |  |  |  |  | 56 | 2611 |

**Table S3,** continuation**.** Calculated Ag and Au concentrations in different fluid inclusion types from 9907 m depth.

| **9907** | | | | | | | |
| --- | --- | --- | --- | --- | --- | --- | --- |
| **L-V-Halite** | | **L-V** | | **V-L** | | **LVCO_2_+L** | |
| Ag ppm | Au ppm | Ag ppm | Au ppm | Ag ppm | Au ppm | Ag ppm | Au ppm |
|  |  |  |  |  |  |  |  |
| 9 | 55 | 161 | 573 | 6 | 54 | 2682 | 4836 |
| 5 |  |  | 145 | 2 | 8 | 110 | 2577 |
| 18 | 352 | 43 | 136 |  | 6 | 542 | 1796 |
| 11 | 35 | 13 | 5 | 11 | 35 | 937 | 8081 |
| 14 | 10 | 12 | 4 | 7 | 326 | 6755 | 191 |
|  | 125 | 75 | 634 | 5 | 51 | 49 | 158 |
| 34 | 14 |  | 585 | 1 | 6 | 265 | 31 |
| 14 | 20 | 66 | 192 | 4 | 5 | 63 | 69 |
|  | 42 | 115 | 45 | 6 | 31 | 433 | 26 |
| 8 | 94 | 93 | 691 | 54 | 204 | 34 | 646 |
| 21 |  |  | 302 | 20 | 60 | 241 | 16 |
| 42 | 832 | 79 | 190 | 2 | 7 | 402 | 3211 |
| 114 | 551 | 3 |  | 21 | 256 | 17 | 29 |
| 4 |  | 17 | 97 | 10 | 8 | 35103 | 5115 |
| 14 | 682 |  | 168 |  |  | 11314 | 4121 |
| 13 | 50 | 43 |  |  |  | 265808 | 21303 |
| 282 | 1779 | 51 | 136 |  |  | 41681 | 27 |
| 8 | 18 |  |  |  |  |  |  |
| 50 | 353 |  |  |  |  |  |  |
| 46 |  |  |  |  |  |  |  |
| 63 | 351 |  |  |  |  |  |  |
| 20 | 73 |  |  |  |  |  |  |


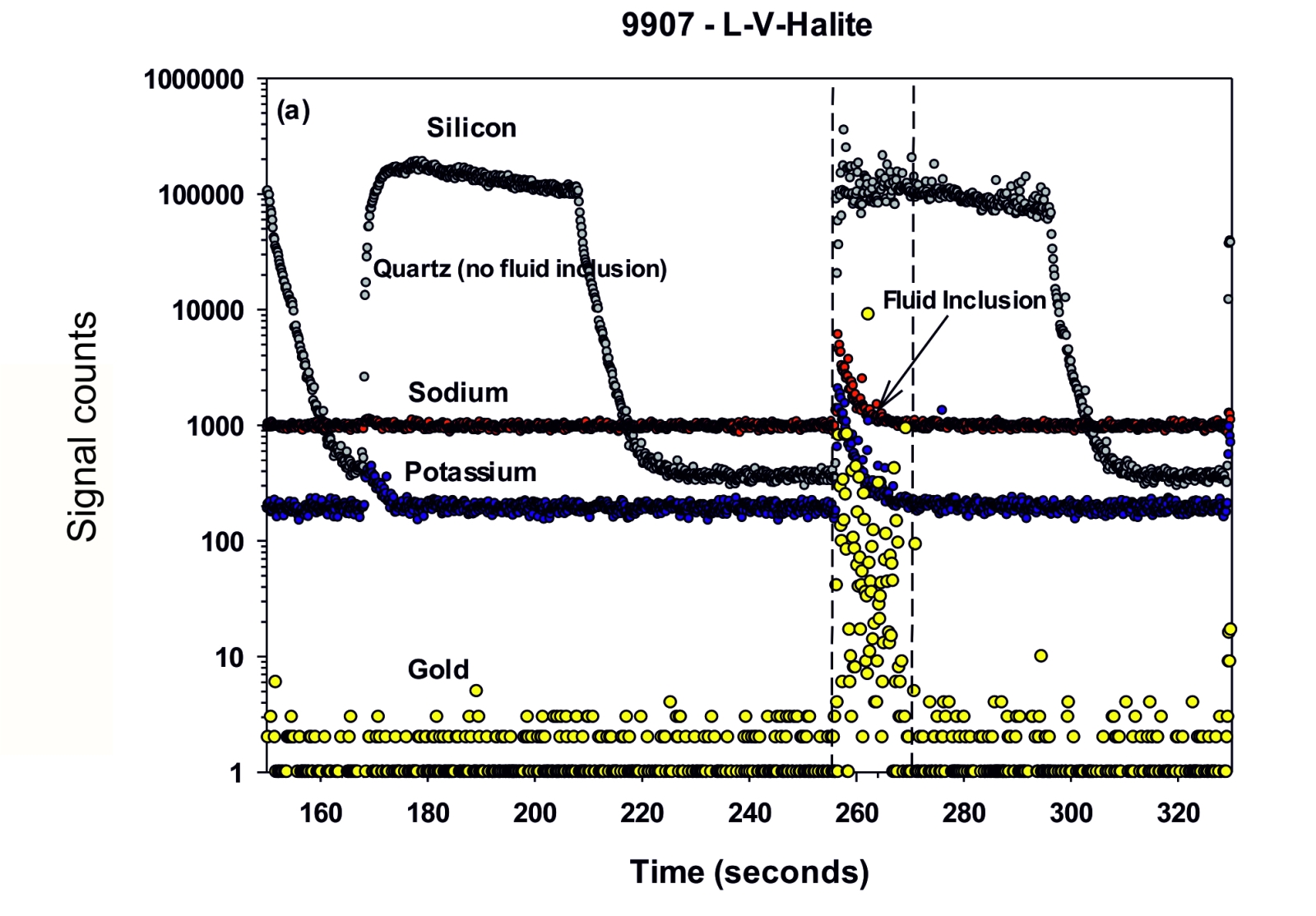


Figure S2. LA-ICP-MS of quartz without fluid inclusions and with fluid inclusions. It can be seen that gold is present only when the fluid inclusion is ablated: Au signal is absent from the quartz and it only appears during the period when there is a signal from the inclusion (K-Na signal).


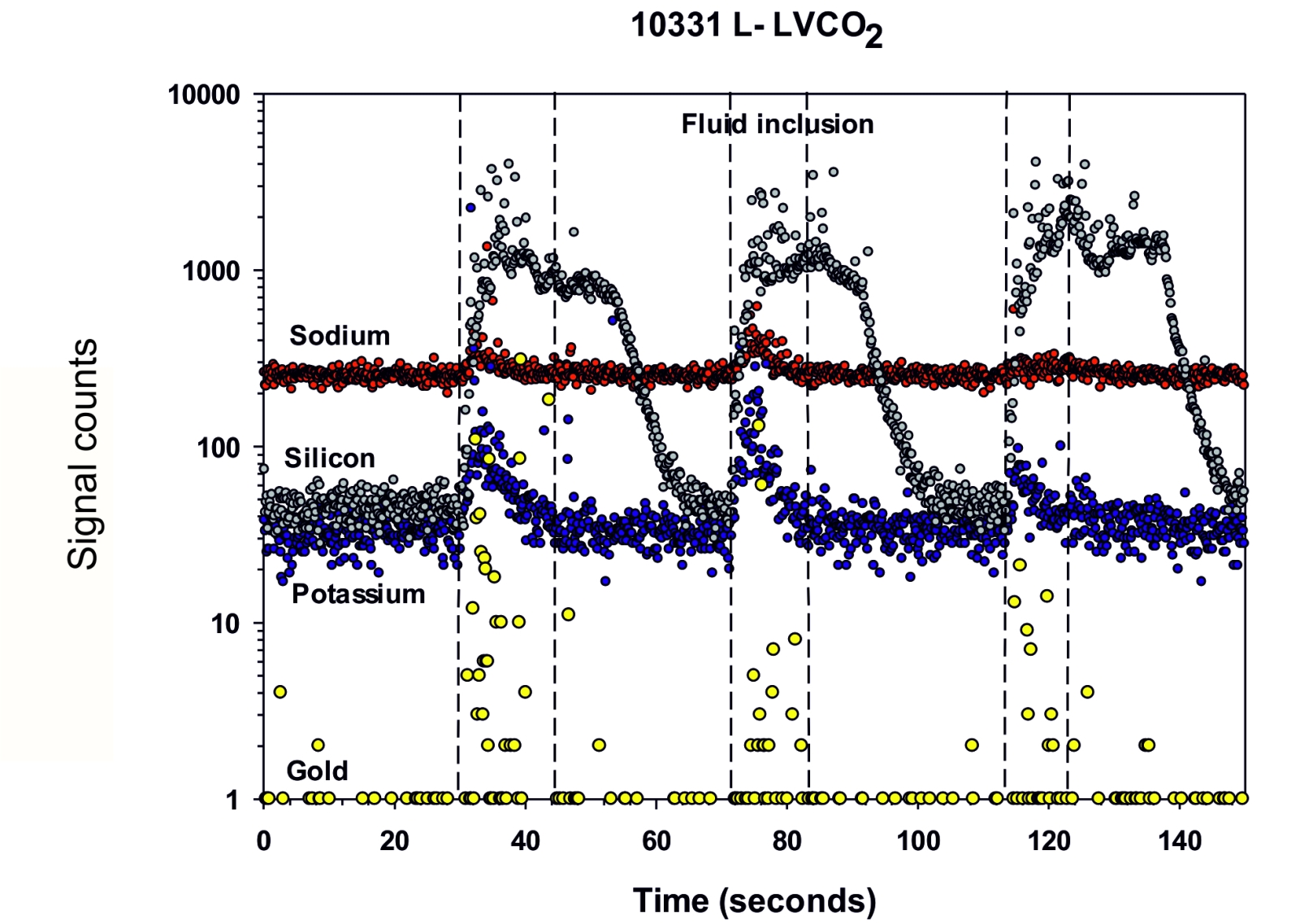


Figure S3. LA-ICP-MS of a series of fluid inclusions that show gold is only present in inclusions. Continued ablation after the inclusion is emptied shows no gold is present in the quartz below the inclusion.


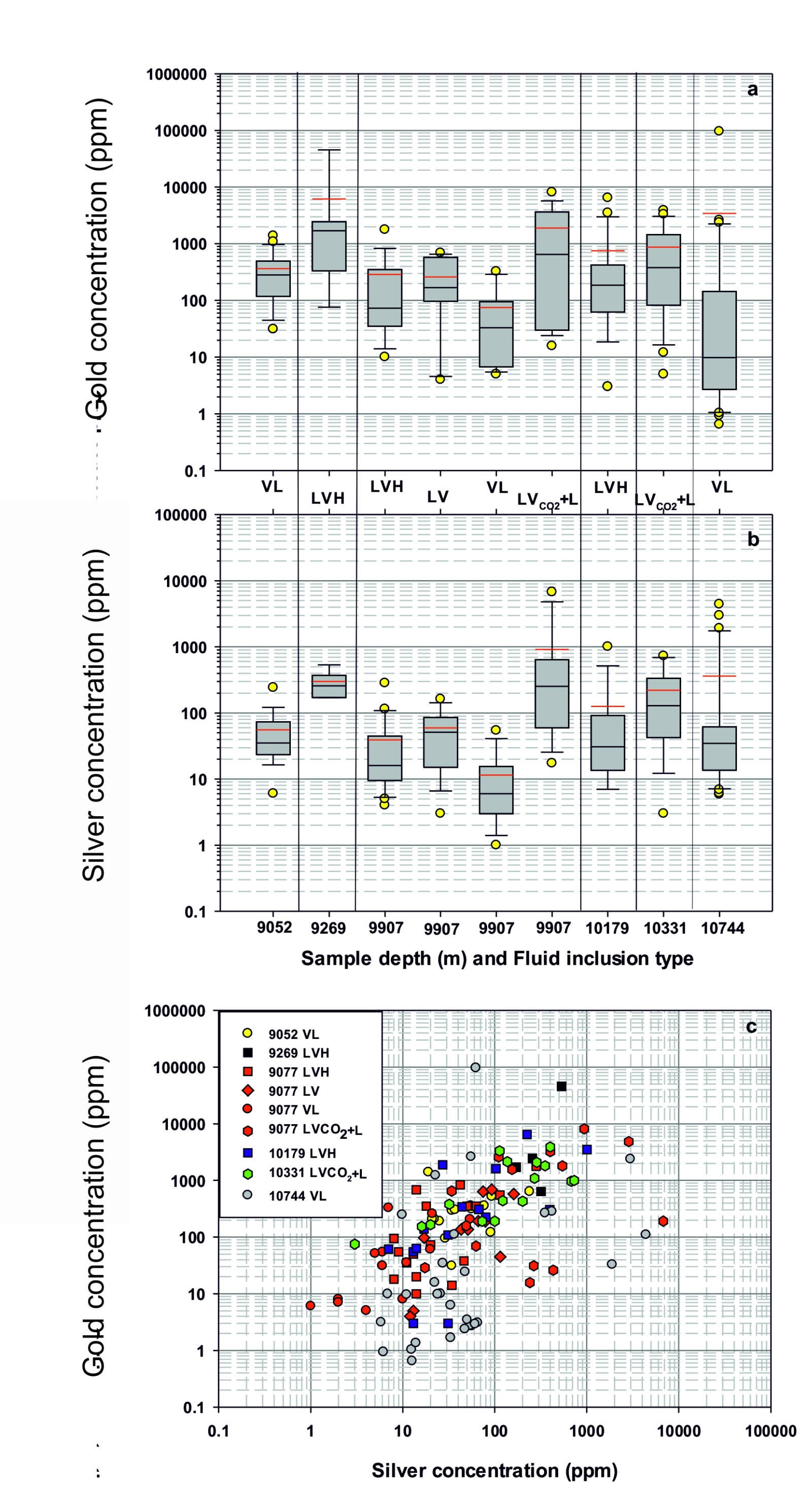


Figure S4. Boxplot diagrams for the gold concentrations (a) and silver concentrations (b), and gold concentrations vs silver concentrations diagram (c).

**Table S4.** **Sample Depth 9907 m. (L-V aqueous inclusions, salinity c. 28% NaCl equiv.)** The inclusions were analyzed using 2 different analytical programs with some elements in common and the results combined. One program consisted of elements to give a general analyses of the fluid inclusions, the other concentrated more on getting the Ag and Au analyses.

|  |  |  |  |  |  |  |  |  |  |  |  |  |  |  |  |
| --- | --- | --- | --- | --- | --- | --- | --- | --- | --- | --- | --- | --- | --- | --- | --- |
| **Type** | **Li^7^** | **Na^23^** | **Mg^24^** | **K39** | **Ca^40^** | **Mn^55^** | **Fe^56^** | **Cu^63^** | **Zn^66^** | **Sr^88^** | **Ag^107^** | **Ba^137^** | **Pb^208^** | **Sb^121^** | **Au^197^** |
|  |  |  |  |  |  |  |  |  |  |  |  |  |  |  |  |
| **L-V** | 0.0209 | 1.3582 | 0.0324 | 1 | 2.3883 | 0.0248 | 0.0319 | 0.0199 | 0.0404 | 0.0523 | 0.0002 |  | 0.0051 |  |  |
|  | 0.0269 | 1.4430 | 0.0251 | 1 | 2.8155 | 0.0284 | 0.0327 | 0.0163 | 0.0414 | 0.0612 | 0.0002 |  | 0.0048 |  |  |
|  | 0.0239 | 1.4448 |  | 1 | 1.3267 | 0.0181 |  |  |  | 0.0340 |  | 0.0191 |  |  |  |
|  | 0.0353 |  |  | 1 | 2.4658 | 0.0180 | 0.0476 | <0.005 | 0.0168 | 0.0713 | <0.0001 | 0.0053 | 0.0061 |  |  |
|  | 0.0220 | 2.1779 |  | 1 | 2.7618 | 0.0332 | 0.0168 | 0.0042 | 0.0177 | 0.0747 | <0.000 | 0.0053 | 0.0064 |  |  |
|  | 0.0235 | 2.1527 | 0.0597 | 1 | 3.0500 | 0.0111 | 0.0075 | 0.0037 |  | 0.0524 |  | 0.0052 | 0.0022 |  |  |
|  | <0.007 | 0.5957 | 0.0448 | 1 | 0.9592 | 0.0019 | 0.0803 |  | 0.0590 | 0.0011 | 0.0004 | 0.0020 | 0.0052 |  |  |
|  | 0.0492 |  |  | 1 |  | 0.0166 |  | 0.0203 | 0.0261 |  | 0.000 |  | 0.0069 |  |  |
|  | 0.0079 | 1.0286 | 0.0918 | 1 | 2.6452 | 0.0090 | 0.0096 | 0.0101 | 0.0095 | 0.0482 |  | 0.0155 | 0.0020 |  |  |
|  | <0.010 | 0.3572 | 0.0763 | 1 |  | <0.002 | 0.0227 |  |  |  | <0.0002 | <0.001 | 0.0038 |  |  |
|  | <0.032 |  |  | 1 |  | 0.0152 | 0.0055 | <0.017 | 0.0216 |  | <0.0006 |  | 0.0046 |  |  |
|  | <0.021 |  |  | 1 |  | <0.004 | 0.0547 | 0.0464 | 0.0325 |  | <0.0004 | 0.0023 | 0.0057 |  |  |
|  | 0.0105 | 0.4067 | 0.0393 | 1 | 1.1959 | <0.002 | 0.0311 | 0.0270 | 0.0217 | 0.0285 | <0.0002 | 0.0082 | 0.0021 |  |  |
|  | <0.071 | 1.0911 |  | 1 | 0.5690 | <0.015 | 0.0744 | 0.0539 | 0.0634 | 0.0037 | <0.0014 | <0.007 | 0.0024 |  |  |
|  |  | 0.7995 |  | 1 |  | <0.010 | 0.0225 | 0.0401 | 0.0479 |  | 0.0010 |  | 0.0037 |  |  |
|  | 0.0137 | 0.5437 |  | 1 | 3.2949 | <0.001 | 0.0157 | 0.0210 | 0.0212 | 0.0576 | <0.0001 | 0.0154 | 0.0016 |  |  |
|  | 0.0308 | 2.4096 |  | 1 |  | 0.0167 | 0.0165 | 0.0125 | 0.0314 |  | 0.0004 |  | 0.0041 |  |  |
|  |  |  |  |  |  |  |  |  |  |  |  |  |  |  |  |
|  |  | 2.0389 |  | 1 |  |  |  |  |  |  | 0.0069 |  |  |  | 0.0248 |
|  |  |  |  | 1 |  |  |  |  |  |  | <0.0011 |  |  | <0.002 | 0.0063 |
|  |  |  |  | 1 |  |  |  |  |  |  | 0.0019 |  |  | <0.0004 | 0.0059 |
|  |  |  |  | 1 |  |  |  |  |  |  | 0.0006 |  |  | <0.0003 | 0.0002 |
| **L-V** |  |  | 0.1185 | 1 |  |  |  |  |  |  | 0.0005 |  |  | <0.0003 | 0.00021 |
|  |  |  |  | 1 |  |  |  |  |  |  | <0.0019 |  |  | <0.0033 | <0.001 |
|  |  |  | 0.0267 | 1 |  |  |  |  |  |  | <0.0002 |  |  | 0.00049 | <0.0001 |
|  |  | 2.4039 |  | 1 |  |  |  |  |  |  | 0.0033 |  |  | <0.0005 | 0.0274 |
|  |  | 2.3324 |  | 1 |  |  |  |  |  |  |  |  |  | <0.0016 | 0.0253 |
|  |  | 0.5762 |  | 1 |  |  |  |  |  |  | 0.0029 |  |  |  | 0.0083 |
|  |  | 0.3082 |  | 1 |  |  |  |  |  |  | 0.0050 |  |  | 0.0027 | 0.00197 |
|  |  | 2.5734 |  | 1 |  |  |  |  |  |  | 0.0040 |  |  | 0.0019 | 0.02997 |
|  |  | 2.6033 |  | 1 |  |  |  |  |  |  | <0.004 |  |  | <0.0032 | 0.0131 |
|  |  | 2.6386 |  | 1 |  |  |  |  |  |  | 0.0034 |  |  | <0.0012 | 0.0083 |
|  |  | 0.5363 | 0.0127 | 1 |  |  |  |  |  |  | 0.0002 |  |  | 0.0008 | <0.0001 |
|  |  |  | 0.0465 | 1 |  |  |  |  |  |  | <0.0003 |  |  | 0.0004 | <0.0002 |
|  |  | 0.5836 | 0.0454 | 1 |  |  |  |  |  |  | <0.0006 |  |  | 0.0008 | <0.0004 |
|  |  | 1.2228 |  | 1 |  |  |  |  |  |  | 0.0008 |  |  | 0.0055 | 0.0042 |
|  |  | 0.4653 | 0.0823 | 1 |  |  |  |  |  |  | <0.0001 |  |  | 0.0010 | <0.0005 |
|  |  |  |  | 1 |  |  |  |  |  |  | <0.0010 |  |  | <0.000 | 0.0073 |
|  |  | 1.3101 | 0.0674 | 1 |  |  |  |  |  |  | 0.0019 |  |  | 0.0011 |  |
|  |  |  |  | 1 |  |  |  |  |  |  | 0.0023 |  |  | 0.0002 | 0.0059 |
|  |  |  |  |  |  |  |  |  |  |  |  |  |  |  |  |
| **Average** | 0.0241 | 1.3616 | 0.0546 | 1 | 2.1338 | 0.0175 | 0.0313 | 0.0229 | 0.0322 | 0.0441 | 0.00191 | 0.0087 | 0.0042 | 0.00153 | 0.0113 |
| **Std Dev** | 0.0117 | 0.8257 | 0.0298 | 0 | 0.9403 | 0.0088 | 0.0233 | 0.0161 | 0.0162 | 0.0247 | 0.0019 | 0.0063 | 0.0017 | 0.0016 | 0.0103 |
| **% RSD** | 48.7 | 60.6 | 54.7 | 0 | 44.1 | 50.3 | 74.4 | 70.4 | 50.4 | 56.1 | 100.6 | 72.3 | 40.5 | 104.5 | 91.4 |

**Table S4,** continuation**. Sample Depth 9907 m. (V-rich aqueous inclusions, salinity c. 3.7 NaCl equiv.)** The inclusions were analysed using 2 different analytical programs with some elements in common and the results combined. One program consisted of elements to give a general analyses of the fluid inclusions, the other concentrated more on getting the Ag and Au analyses.

| **Type** | **Li^7^** | **Na^23^** | **Mg^24^** | **K39** | **Ca^40^** | **Mn^55^** | **Fe^56^** | **Cu^63^** | **Zn^66^** | **Sr^88^** | **Ag^107^** | **Ba^137^** | **Pb^208^** | **Sb^121^** | **Au^197^** |
| --- | --- | --- | --- | --- | --- | --- | --- | --- | --- | --- | --- | --- | --- | --- | --- |
|  |  |  |  |  |  |  |  |  |  |  |  |  |  |  |  |
| **V-rich** | <0.2441 | 0.8295 | 0.01493 | 1 |  | <0.0435 |  | <0.1226 | 0.0971 |  | <0.0016 | <0.0255 | <0.0065 |  |  |
|  | <0.1960 | 2.4934 |  | 1 | 0.4187 | <0.0350 | 0.1708 | <0.0981 | 0.0802 | <0.0010 | 0.0022 | <0.0206 | 0.0076 |  |  |
|  | <0.1688 |  |  | 1 | 0.3379 | <0.0301 | 0.0554 | <0.0825 | 0.0664 | 0.0013 | 0.0021 | <0.0176 | <0.0046 |  |  |
|  | <0.0555 | 0.6498 | 0.0243 | 1 | 0.4578 | <0.0100 |  | 0.10281 | 0.1402 |  | 0.0007 | 0.0172 |  |  |  |
|  |  |  | 0.1035 | 1 |  |  |  |  |  | <0.0065 | <0.0060 |  |  |  |  |
|  |  | 1.8798 |  | 1 |  |  |  | <0.2521 | 0.1728 | 0.0043 |  |  | <0.0148 |  |  |
|  | <0.0748 |  | 0.0939 | 1 | 0.1798 | <0.0140 | 0.0510 | 0.0401 | 0.0494 | 0.0069 | 0.0005 | 0.0138 | 0.0095 |  |  |
|  | <0.3729 |  |  | 1 | <0.0641 | <0.0703 | <0.0471 | <0.1810 | <0.038 | 0.00424 | 0.00712 | <0.0402 | <0.0109 |  |  |
|  | <0.3568 | 1.1602 | 0.1047 | 1 | 0.6701 | <0.0640 | 0.1366 | <0.1702 | 0.1783 | <0.0021 | 0.0024 | <0.0384 |  |  |  |
|  | <0.2006 |  | 0.0272 | 1 | 0.3195 | <0.0341 | 0.0892 | <0.1006 | 0.1124 | 0.0022 | <0.0012 | <0.0202 | <0.0053 |  |  |
|  | <0.3447 |  | <0.0252 | 1 |  | <0.0633 | 0.09886 | <0.1678 | 0.10817 | 0.00228 | 0.00469 |  | <0.0097 |  |  |
|  |  |  |  |  |  |  |  |  |  |  |  |  |  |  |  |
|  |  | 0.5647 |  | 1 |  |  |  |  |  |  | 0.0014 |  |  | 0.0034 | 0.0119 |
|  |  | 0.5728 |  | 1 |  |  |  |  |  |  | 0.0005 |  |  | 0.0006 | 0.0018 |
|  |  | 1.4417 |  | 1 |  |  |  |  |  |  | <0.0004 |  |  | <0.0007 | 0.0013 |
|  |  | 1.0092 |  | 1 |  |  |  |  |  |  | 0.0024 |  |  | 0.0021 | 0.0077 |
|  |  | 1.3061 |  | 1 |  |  |  |  |  |  | 0.0015 |  |  | 0.00536 | 0.0712 |
|  |  | 1.2529 |  | 1 |  |  |  |  |  |  | 0.0012 |  |  | 0.0016 | 0.0112 |
|  |  |  |  | 1 |  |  |  |  |  |  | 0.0002 |  |  | <0.0001 | 0.0014 |
|  |  | 1.4624 |  | 1 |  |  |  |  |  |  | 0.0010 |  |  | 0.0005 | 0.0011 |
|  |  | 1.3664 |  | 1 |  |  |  |  |  |  | 0.0013 |  |  | 0.0010 | 0.0069 |
|  |  | 1.0418 |  | 1 |  |  |  |  |  |  | 0.0118 |  |  | <0.0040 | 0.0446 |
|  |  | 1.3140 |  | 1 |  |  |  |  |  |  | 0.0045 |  |  | <0.0013 | 0.0132 |
| **V-rich** |  | 0.69032 |  | 1 |  |  |  |  |  |  | 0.00057 |  |  | 0.0015 | 0.0017 |
|  |  | 2.2193 |  | 1 |  |  |  |  |  |  | 0.0045 |  |  | 0.0001 | 0.0559 |
|  |  | 1.0420 |  | 1 |  |  |  |  |  |  | 0.0022 |  |  | 0.0041 | 0.00190 |
|  |  |  |  |  |  |  |  |  |  |  |  |  |  |  |  |
| **Average** | 0.04300 | 1.2387 | 0.0614 | 1 | 0.3973 | 0.0405 | 0.1003 | 0.0715 | 0.1116 | 0.0035 | 0.0026 | 0.0242 | 0.0086 | 0.00215 | 0.0166 |
| **Std Dev** |  | 0.5376 | 0.0433 | 0 | 0.1645 | 0.0217 | 0.0465 | 0.0442 | 0.0449 | 0.0020 | 0.00280 | 0.0099 | 0.0033 | 0.0016 | 0.0230 |
| **% RSD** |  | 43.4 | 70.6 | 0 | 41.4 | 53.6 | 46.4 | 61.9 | 40.2 | 57.7 | 104.8 | 41.0 | 38.5 | 76.2 | 138.9 |

**Table S4,** continuation**. Sample Depth 9907 m. (L-V-Halite aqueous inclusions, salinity c. 31% NaCl equiv.)** The inclusions were analysed using 2 different analytical programs with some elements in common and the results combined. One program consisted of elements to give a general analyses of the fluid inclusions, the other concentrated more on getting the Ag and Au analyses.

| **Type** | **Li^7^** | **Na^23^** | **Mg^24^** | **K^39^** | **Ca^40^** | **Mn^55^** | **Fe^56^** | **Cu^63^** | **Zn^66^** | **Sr^88^** | **Ag^107^** | **Ba^137^** | **Pb^208^** | **Sb^121^** | **Au^197^** |
| --- | --- | --- | --- | --- | --- | --- | --- | --- | --- | --- | --- | --- | --- | --- | --- |
|  |  |  |  |  |  |  |  |  |  |  |  |  |  |  |  |
| **L-V-H** | <0.0345 | 0.8951 | 0.0836 | 1 | 0.3981 | <0.0067 | 0.0565 |  | 0.0552 | 0.0008 | 0.0041 | 0.0874 | 0.0056 |  |  |
|  |  | 0.9163 |  | 1 | 0.1084 | <0.0245 | 0.0299 |  |  | <0.0007 | <0.0028 | <0.0116 | <0.0038 |  |  |
|  | <0.0145 | 1.2881 |  | 1 | 0.6017 | <0.0029 | 0.0429 | 0.1036 | 0.0291 | 0.0007 | 0.0051 | <0.0013 | 0.0014 |  |  |
|  |  | 0.4994 |  | 1 | 0.7388 |  |  | 0.0919 |  | 0.0163 | 0.0039 | 0.0461 |  |  |  |
|  | <0.0387 |  | 0.0649 | 1 |  | 0.0072 |  | 0.0702 |  |  | 0.0009 |  | 0.0060 |  |  |
|  | <0.0222 | 0.7318 |  | 1 | 0.5207 | 0.0067 |  | 0.0527 | 0.0993 | 0.0227 | 0.0010 | 0.0029 | 0.0064 |  |  |
|  |  | 2.0349 |  | 1 | 0.3660 | <0.0087 |  | 0.1080 |  | 0.0023 | 0.0029 |  |  |  |  |
|  | <0.0049 |  | 0.0314 | 1 |  | <0.0008 | 0.0174 | 0.0529 | 0.0112 | 0.0002 | 0.0002 |  | 0.0012 |  |  |
|  | <0.0194 |  |  | 1 |  |  | 0.0720 | 0.0118 | 0.0497 |  | <0.0002 |  | 0.0044 |  |  |
|  |  | 1.4931 | 0.0700 | 1 |  |  | 0.0952 | 0.0841 | 0.0789 |  | 0.0015 |  |  |  |  |
|  |  | 0.6299 |  | 1 |  | 0.0008 | 0.0336 | 0.0254 | 0.0074 | 0.0009 | 0.00004 |  | 0.0009 |  |  |
|  | <0.0230 | 0.5291 | 0.0314 | 1 |  |  | 0.0477 | 0.0328 | 0.0313 |  | <0.0003 | 0.0640 |  |  |  |
|  | 0.0287 | 1.5149 | 0.0357 | 1 |  | 0.0088 | 0.0192 | 0.0186 | 0.0161 |  | 0.0004 |  | 0.0048 |  |  |
|  | 0.0048 | 0.6630 |  | 1 | 0.7474 | 0.0037 | 0.0403 |  | 0.0388 | 0.0529 | 0.0001 | 0.0085 | 0.0034 |  |  |
|  | 0.0158 | 1.9628 |  | 1 |  | 0.0055 | 0.0380 | 0.0416 | 0.0239 |  | 0.0002 | 0.0767 | 0.0035 |  |  |
|  | 0.0519 | 0.4810 |  | 1 |  |  | 0.0414 | 0.0269 | 0.0673 | 0.0761 | <0.0002 | 0.0373 |  |  |  |
|  |  |  |  |  |  |  |  |  |  |  |  |  |  |  |  |
|  |  | 1.4665 | 0.0562 | 1 |  |  |  |  |  |  | 0.0002 |  |  | <0.0002 | 0.0014 |
|  |  | 1.2804 | 0.0118 | 1 |  |  |  |  |  |  | 0.0001 |  |  | 0.0010 | <0.0001 |
|  |  | 0.8445 | 0.0723 | 1 |  |  |  |  |  |  | <0.0002 |  |  | <0.0003 | <0.0001 |
|  |  | 2.2203 |  | 1 |  |  |  |  |  |  | 0.0004 |  |  | <0.0002 | 0.0087 |
|  |  | 0.9566 | 0.0163 | 1 |  |  |  |  |  |  | <0.0006 |  |  | <0.0008 | <0.0003 |
|  |  | 0.8163 | 0.0177 | 1 |  |  |  |  |  |  | 0.0003 |  |  | 0.0017 | 0.0009 |
| **L-V-H** |  | 0.9732 | 0.0418 | 1 |  |  |  |  |  |  | 0.0004 |  |  | 0.0003 | 0.0003 |
|  |  | 0.5697 |  | 1 |  |  |  |  |  |  | <0.0005 |  |  | <0.0007 | 0.0031 |
|  |  |  |  | 1 |  |  |  |  |  |  | 0.0009 |  |  | <0.0004 | 0.0035 |
|  |  | 1.4885 |  | 1 |  |  |  |  |  |  | 0.0004 |  |  | <0.0001 | 0.0005 |
|  |  | 2.3097 |  | 1 |  |  |  |  |  |  | <0.0002 |  |  | <0.0003 | 0.0010 |
|  |  | 2.7817 | 0.0880 | 1 |  |  |  |  |  |  | 0.0002 |  |  | <0.0004 | 0.0023 |
|  |  | 1.4411 | 0.029 | 1 |  |  |  |  |  |  | 0.0005 |  |  | <0.0009 | <0.0004 |
|  |  | 1.5848 |  | 1 |  |  |  |  |  |  | 0.0011 |  |  | <0.001 | 0.0206 |
|  |  | 0.5875 |  | 1 |  |  |  |  |  |  | 0.0028 |  |  | <0.001 | 0.0136 |
|  |  |  |  | 1 |  |  |  |  |  |  | 0.0001 |  |  | <0.0001 | <0.0001 |
|  |  |  |  | 1 |  |  |  |  |  |  | 0.0004 |  |  | <0.0005 | 0.0169 |
|  |  | 1.3169 |  | 1 |  |  |  |  |  |  | 0.0003 |  |  | 0.0002 | 0.0012 |
|  |  | 0.4951 |  | 1 |  |  |  |  |  |  | 0.0070 |  |  | 0.0008 | 0.0441 |
|  |  | 2.5394 |  | 1 |  |  |  |  |  |  | 0.0002 |  |  | 0.0002 | 0.0005 |
|  |  | 0.6934 |  | 1 |  |  |  |  |  |  | 0.0012 |  |  | 0.0008 | 0.0087 |
|  |  | 1.5370 |  | 1 |  |  |  |  |  |  | 0.0011 |  |  | <0.0005 | 0.0001 |
|  |  | 0.6174 |  | 1 |  |  |  |  |  |  | 0.0015 |  |  | <0.0003 | 0.0087 |
|  |  |  |  | 1 |  |  |  |  |  |  | 0.0005 |  |  | <0.0002 | 0.0018 |
|  |  |  |  |  |  |  |  |  |  |  |  |  |  |  |  |
| **Average** | 0.0234 | 1.2171 | 0.0464 | 1 | 0.4974 | 0.0054 | 0.0445 | 0.0554 | 0.0423 | 0.0192 | 0.0012 | 0.0462 | 0.0038 | 0.0007 | 0.0073 |
| **Std Dev** | 0.0142 | 0.6431 | 0.0257 | 0. | 0.2275 | 0.0028 | 0.0218 | 0.0330 | 0.0285 | 0.0275 | 0.0016 | 0.0324 | 0.0020 | 0.0005 | 0.0108 |
| **% RSD** | 60.8 | 52.8 | 55.3 | 0 | 45.7 | 51.8 | 49.1 | 59.7 | 67.3 | 142.9 | 132.1 | 70.2 | 53.3 | 77.1 | 147.3 |

**Table S4,** continuation**. Sample Depth 9907 m. (Type 4 LVCO_2_ + L aqueous inclusions, average salinity c. 6% NaCl equiv. based on the clathrate melting temperature).**

| **Sample** | **Type** | **Na^23^** | **Mg^24^** | **K^39^** | **Ca^40^** | **Ag^107^** | **Au^197^** |
| --- | --- | --- | --- | --- | --- | --- | --- |
|  |  |  |  |  |  |  |  |
| 9907-a | **LV CO_2_+L** | 1.9174 |  | 1 | 1.3612 | .0134 | .3128 |
|  |  |  |  | 1 |  | 4.261 | .6208 |
|  |  |  |  | 1 |  | .0658 | .2180 |
|  |  |  |  | 1 |  | .1137 | .9809 |
|  |  | 1.7997 |  | 1 |  | .8199 | .0231 |
|  |  | 1.2011 |  | 1 |  | .0059 | .0192 |
|  |  | 1.8448 |  | 1 |  | 1.373 | .5003 |
|  |  | 1.4112 |  | 1 |  | 32.266 | 2.585 |
| 9907-b |  |  | .4943 | 1 |  | .0321 | .0038 |
|  |  | 1.2641 |  | 1 |  | .0076 | .0084 |
|  |  | 1.0162 | .4137 | 1 | .5823 | .0525 | .0032 |
|  |  | .7676 |  | 1 | .9166 | 5.059 | .0033 |
|  |  | 2.2916 | .3696 | 1 |  | .0041 | .0784 |
| 9907-c |  | 1.2951 | .0365 | 1 | 1.3316 | .0295 | .0019 |
|  |  | .7676 | .1101 | 1 | 1.3652 | .0487 | .3898 |
|  |  |  |  | 1 |  | .0021 | .0035 |
|  |  | 1.7520 |  | 1 |  | .0187 | .1883 |
|  |  | .5209 |  | 1 | 1.8675 | .3473 | .5870 |
|  |  |  |  |  |  |  |  |
| Average |  | 1.3730 | .2849 | 1 | 1.2374 | 2.4734 | .3627 |
| Std Dev |  | .5263 | .1999 | 0 | .4404 | 7.8264 | .6232 |
| %RSD |  | 38.3 | 70.2 | 0 | 35.6 | 306.6 | 171.8 |
